# Association between human blood metabolites and cerebral cortex architecture: evidence from a Mendelian randomization study

**DOI:** 10.3389/fneur.2024.1386844

**Published:** 2024-05-09

**Authors:** Zongzhi Jiang, Yining Sun, Songyan Liu

**Affiliations:** ^1^Department of Neurology, China-Japan Union Hospital, Jilin University, Changchun, China; ^2^Department of Neurology, The First Hospital of Jilin University, Changchun, China

**Keywords:** brain cortex thickness, brain cortex surficial area, metabolites, genome-wide association studies, Mendelian randomization

## Abstract

**Background:**

Dysregulation of circulating metabolites may affect brain function and cognition, associated with alterations in the cerebral cortex architecture. However, the exact cause remains unclear. This study aimed to determine the causal effect of circulating metabolites on the cerebral cortex architecture.

**Methods:**

This study utilized retrieved data from genome-wide association studies to investigate the relationship between blood metabolites and cortical architecture. A total of 1,091 metabolites and 309 metabolite ratios were used for exposure. The brain cortex surface area and cortex thickness were selected as the primary outcomes in this study. In this study, the inverse variance weighting method was used as the main analytical method, complemented by sensitivity analyses that were more robust to pleiotropy. Furthermore, metabolic pathway analysis was performed via MetaboAnalyst 6.0. Finally, reverse Mendelian randomization (MR) analysis was conducted to assess the potential for reverse causation.

**Results:**

After correcting for the false discovery rate (FDR), we identified 37 metabolites and 9 metabolite ratios that showed significant causal associations with cortical structures. Among these, Oxalate was found to be most strongly associated with cortical surface area (*β*: 2387.532, 95% CI 756.570–4018.495, *p* = 0.037), while Tyrosine was most correlated with cortical thickness (*β*: −0.015, 95% CI −0.005 to −0.025, *p* = 0.025). Furthermore, pathway analysis based on metabolites identified six significant metabolic pathways associated with cortical structures and 13 significant metabolic pathways based on metabolite ratios.

**Conclusion:**

The identified metabolites and relevant metabolic pathways reveal potential therapeutic pathways for reducing the risk of neurodegenerative diseases. These findings will help guide health policies and clinical practice in treating neurodegenerative diseases.

## Introduction

The cerebral cortex is the high-level center for processing motor and sensory information. High levels of cognitive ability and personality shaping are associated with structure within this cortical region (1). Any structural abnormality within a cortical region can lead to a wide range of neurological and mental disorders. Among them, variations in human cortical surface area and thickness are associated with neurological, psychological, and behavioral traits, emphasizing understanding the factors influencing cortical architecture (2, 3).

In recent years, the study of the relationship between metabolic abnormalities and neurodegenerative diseases has sparked great interest due to the development of snapshotting techniques for the intricate multivariate biochemical processes involved in disease development (4). In the context of an aging population, there has also been much research on the potential link between metabolites and brain anatomy, implying that certain metabolites are involved in developing the cerebral cortex. Kynurenine metabolites, for instance, are strongly associated with reduced right medial prefrontal cortex thickness in major depressive disorder (5). Similarly, Jiang’s et al. (6) study revealed that the N-acetyl-aspartate (NAA)/Creatine (Cr) ratio was significantly lower in patients with reduced hippocampal thickness compared to controls. In addition, a rodent study found a negative correlation between glutamatergic and cortex surficial area and cortex thickness (7). This suggests that glutamatergic metabolites may have neuroprotective effects.

However, potential confounding factors (such as inflammatory factors, tissue proteases, micronutrients, and potential comorbidities) might impact the accuracy and reliability of the conclusions. And, there is still a paucity of comprehensive and systematic research appraising the causal effect of blood metabolites on brain cortical structure. Therefore, clarifying the spectrum of metabolites that lead to alterations in brain cortical structure is crucial for diagnostic and mechanistic studies of neurodegenerative diseases.

Mendelian randomization (MR) applies genetic variation as an instrumental variable (IV) based on Mendelian laws of inheritance to explore the causal relationship between exposure and outcome (8). Thus, the effects of confounding factors in the acquired environment can be overcome and false-negative or false-positive results due to confounding factors and reverse causation in conventional observational studies can be avoided (9). Leveraging genetic variants associated specifically with blood metabolites, we can explore the causal relationship between these metabolites and brain cortical structure more precisely. Given this knowledge gap, we conducted this comprehensive MR research to investigate the influence of blood metabolites on abnormal changes in brain cortical structure. By utilizing data on blood metabolites and neuroimaging from large-scale genome-wide association studies (GWAS), we aim to gain a deeper understanding of the underlying neurobiological mechanisms of metabolite-associated structural brain alterations.

## Materials and methods

### Study design

**Figure 1:**
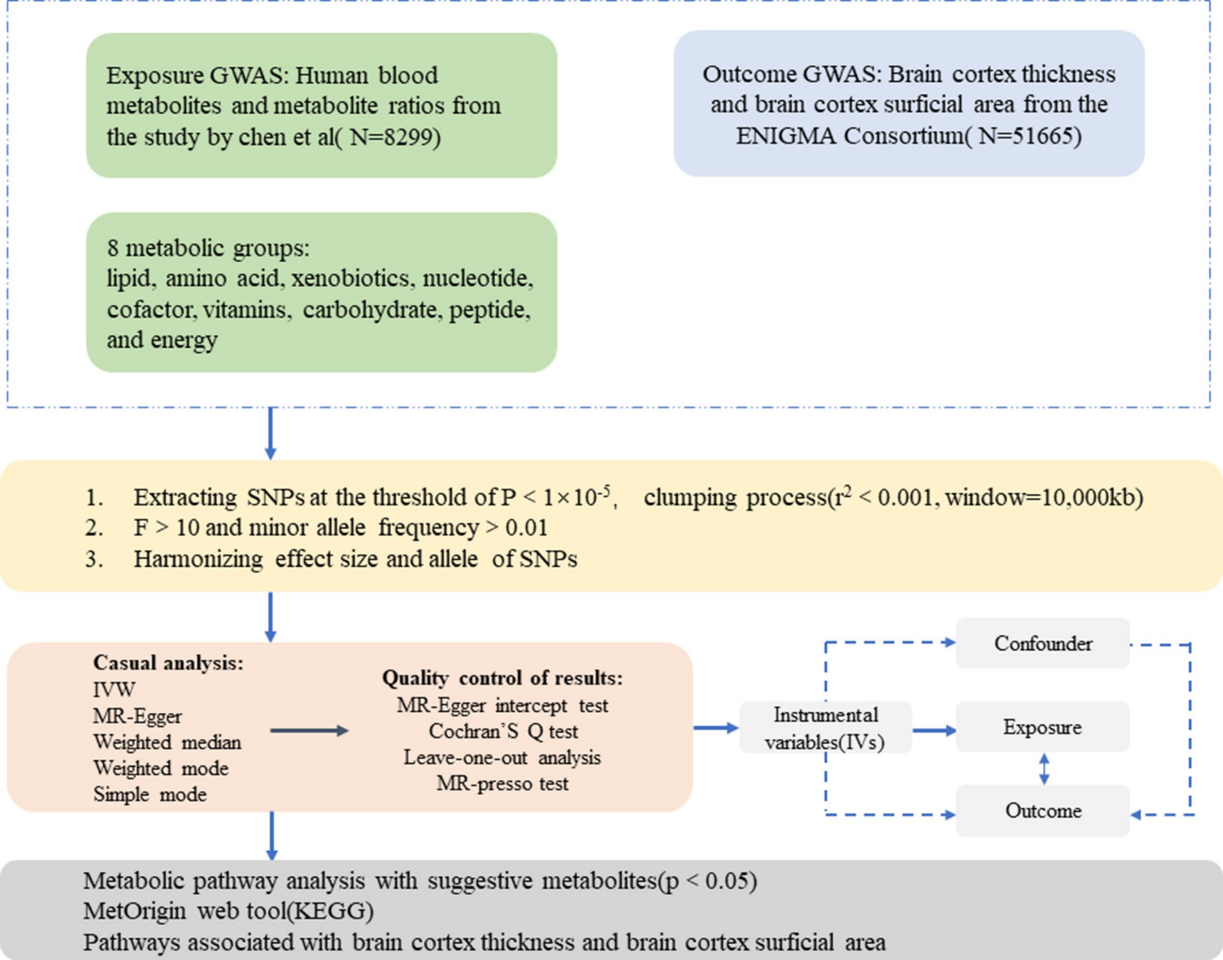
An overall design of the current study. GWAS, genome-wide association study; MR, Mendelian randomization; MR-PRESSO, MR-pleiotropy residual sum and outlier test; SNP, single nucleotide polymorphism; IVs, instrumental variable.

The present study employed an MR design to investigate the causal association between 1,091 blood metabolites, 309 metabolite ratios, and abnormal changes in brain cortical structure. Figure 1 shows the overall design of our two-sample MR study. To obtain credible results from the MR approach, our study tried to satisfy the following three assumptions of an MR study: (1) IVs should be highly correlated with exposure; (2) IVs were not associated with any confounders affecting the exposure-outcome associations; (3) IVs do not directly affect outcome unless aided by association with exposure.

### Data source for human blood metabolites

The GWAS datasets involving 1,091 blood metabolites and 309 metabolite ratios were obtained from the study by Chen et al. (4). This is the currently most comprehensive analysis of plasma metabolome metabolites implicated in human disease, and the full summary statistics of which were publicly available via GWAS Catalog (4).^1^ Eight thousand two hundred ninety-nine European descents were included and approximately 15.4 million Single nucleotide polymorphisms (SNPs) for 1,091 blood metabolites and 309 metabolite ratios were tested. Among the 1,091 metabolites, 850 had known identities. It was assigned to eight broad metabolic groups, including lipid, amino acid, xenobiotics, nucleotide, cofactor, vitamins, carbohydrate, peptide, and energy, as defined in the Kyoto Encyclopedia of Genes and Genomes (KEGG) database (10). The remaining 241 were categorized as unknown or “partially” characterized molecules.

### Data source for brain cortex surficial area and cortex thickness

The GWAS data related to brain cortical structure were obtained from the ENIGMA Consortium (11). Surface area and thickness measurements of the brain cortex were gathered from 51,665 individuals, with a predominant (~94%) European descent, across 60 cohorts worldwide. They identified a set of 8,567,615 SNPs closely related to the brain cortex surficial area. In addition, they also discovered 8,619,054 SNPs showing a significant correlation with cortical thickness.

### Instrumental variables selection

We extracted SNPs strongly associated with metabolites from published data, using *p* < 1 × 10^−5^ as the primary screening condition. To ensure that the instruments used for exposure were independent, we excluded SNPs with linkage disequilibrium (LD) (*r*^2^ < 0.001, clumping window = 10,000 kb). We then extracted the information of instrumental variables from the brain cortical structure GWAS. Palindromic SNPs were excluded. We then harmonized the exposure data with the outcome data, meaning that both the effect of the SNP on exposure and the effect on outcome corresponded to the same allele, thus correcting the strand for non-palindromic SNPs, and removing all palindromic sequences. We also calculated the *F* statistic to eliminate the bias caused by weak instrumental variables in the results. The *F* statistic is calculated as *F* = *R*^2^ (*n* − *k* − 1)/[*k*(1 − *R*^2^)]. *R*^2^ reflects the degree to which the instrumental variable explains the exposure. *N* is the number of exposure samples to the GWAS study and *K* is the number of IVs. An *F*-statistic greater than 10 indicates that weak instrumental variable bias does not affect the results.

### Statistical analysis

In the current study, MR computational models were utilized to determine if there was a causal relationship between blood metabolites and brain cortical structure (cortex surficial area and cortex thickness) using inverse-variance weighted (IVW), MR Egger, weighted median, weighted mode, and simple mode. IVW was used as the primary analysis method in MR studies. IVW is characterized by regressions that do not account for the presence of an intercept term and are fitted. In addition, the MR-Egger method was used as a complementary method to test the robustness of the IVW results. The weighted median is strong in causality detection ability and can be used as a complementary method to test the robustness of its results. Even when up to 50% of the SNPs are invalid IVs, the weighted median tends to robustly yield correct estimates (12). As an additional step, we used weighted mode and simple mode to enhance accuracy and stability. False discovery rate (FDR) correction was used to control for false positives in multiple tests. A statistically significant association was considered if the estimated causal effect of a given metabolite had a FDR <0.05.

We assessed the potential heterogeneity between instrumental variables at each analysis using Cochran’s *Q* test. *p* > 0.05 indicates no significant heterogeneity. We used the MR-Egger intercept to test for horizontal pleiotropy. If its *p*-value is >0.05, it means that there is no horizontal pleiotropy. The Mendelian Randomization Pleiotropy Residual Sum and Outlier (MR-PRESSO) test detects and corrects for horizontal pleiotropy by identifying and removing outliers. Regardless of the various methods we use to ensure the reliability of IVs when screening them, it is still inevitable that some of the extracted IVs are risky for the final result. We therefore used the leave-one-out method for sensitivity analysis to further validate the robustness of the results.

The TSMR analyses in this study were performed using version 4.0.3 of the R software and version 0.5.6 of the “Two Sample MR” package and “MRPRESSO” package. The study was done in R software for mapping^2^.

### Reverse MR analysis

We chose SNPs positive for forward MR analysis as instrumental variables, applied the same workflow for reverse MR analysis, and used IVW as the primary analytical method for MR analysis.

### Metabolic pathway analysis

Using this set of identified metabolites, the final metabolic pathway analysis based on the KEGG database was performed using MetaboAnalyst 6.0,^3^ a user-friendly online tool for simplified metabolomics data analysis (13).

## Results

### Selection of IVs

To explore the causal effect of human blood metabolites as exposure factors on the prevalence of outcomes, 1,400 metabolites (including blood metabolites and metabolite ratios) were retained in the MR estimation (Supplementary Table S1). A total of 3,240 SNPs were found to be associated with the brain cortex surface area (Supplementary Table S2), and 114 SNPs were associated with cortical thickness (Supplementary Table S3). All *F*-statistics were above 10 (Supplementary Tables S2, S3), indicating that the weak instrumental bias was eliminated.

### Causal relationship analysis between blood metabolites, ratios, and brain cortex surficial area

**Figure 2:**
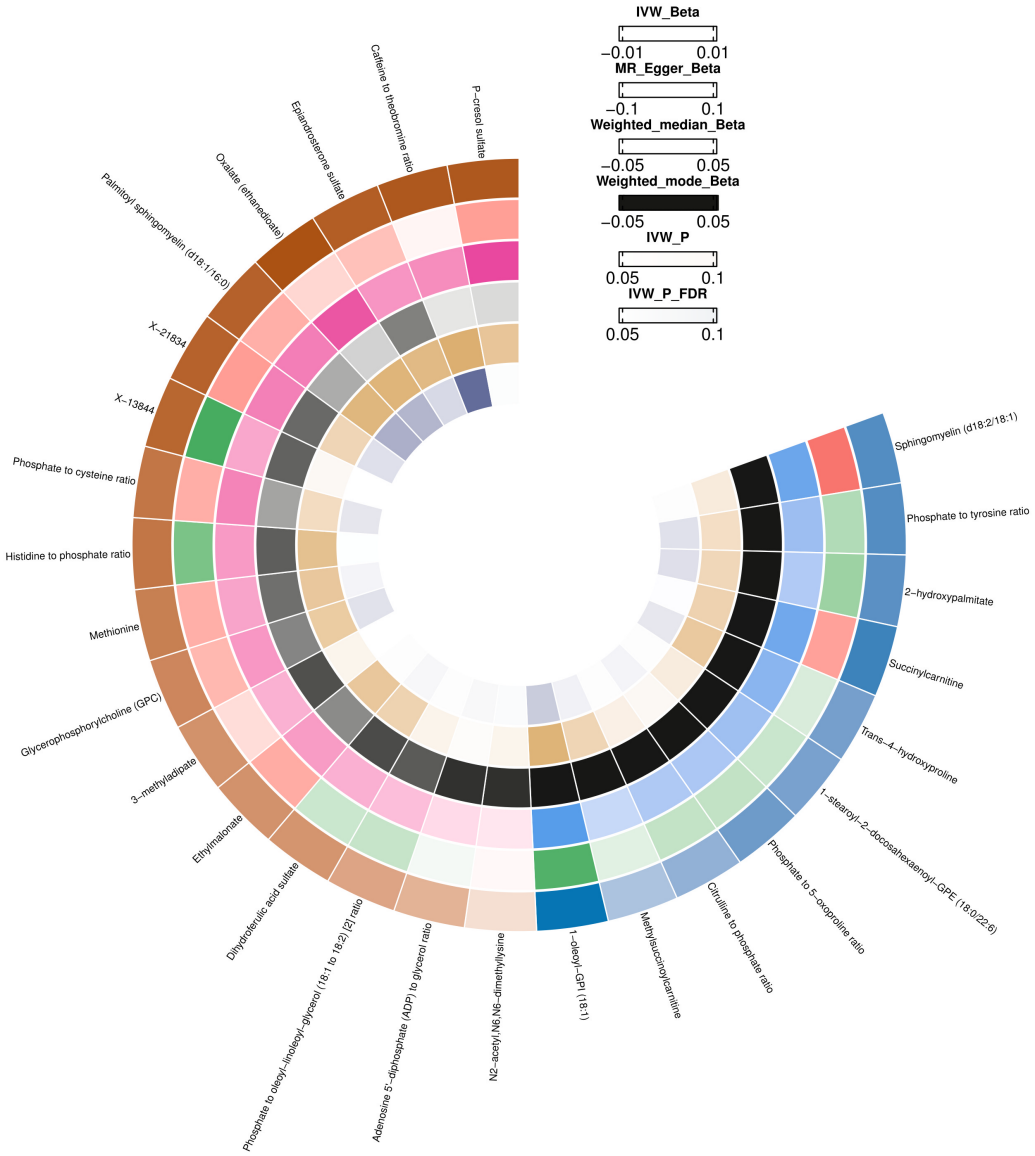
Mendelian randomization associations between blood metabolites and brain cortex surficial area.

Using the IVW method, 18 causal associations with multiple testing-corrected significance (FDR <0.05) were observed. The chemical compositions of these two metabolites remain unknown. Another 16 causal associations included five metabolite ratios and 11 metabolites, which were chemically assigned to nucleotides, lipids, amino acids, energy, xenobiotics, cofactors, and vitamins. They were as follows: Dihydroferulic acid sulfate [*β* = 1430.51, 95% confident intervals (CI): 193.21 to 2667.80, *p* = 0.0234]; Methionine (*β* = 1741.06, CI: 323.62 to 3158.51, *p* = 0.0161); Phosphate to 5-oxoproline ratio (*β* = −1741.54, CI: −3502.18 to −28.89, *p* = 0.0463); Trans-4-hydroxyproline (*β* = −1690.19, CI: −3078.38 to −302.01, *p* = 0.0170); Palmitoyl sphingomyelin (d18:1/16:0) (*β* = 2164.98, CI: 641.84 to 3688.12, *p* = 0.0053); Methylsuccinoylcarnitine (*β* = −1043.10, CI: −1953.69 to −132.51, *p* = 0.0247); Oxalate (ethanedioate) (*β* = 2387.53, CI: 756.57 to 4018.49, *p* = 0.0041); Glycerophosphorylcholine (GPC) (*β* = 1633.06, CI: 275.39 to 2990.72, *p* = 0.0183); 1-oleoyl-GPI (18:1) (*β* = −3200.06, CI: −5316.27 to −1083.84, *p* = 0.0030); N2-acetyl,N6,N6-dimethyllysine (*β* = 427.59, CI: 11.65 to 843.54, *p* = 0.0439); 2-hydroxypalmitate (*β* = −1968.91, CI: −3707.10 to −230.73, *p* = 0.0264); Epiandrosterone sulfate (*β* = 2223.27, CI: 579.10 to 3867.43, *p* = 0.0080); Phosphate to tyrosine ratio (*β* = 2033.92, CI: 3871.04 to −196.81, *p* = 0.0300); Caffeine to theobromine ratio (*β* = 2301.47, CI: 948.68 to 3654.26, *p* = 0.0008); Phosphate to cysteine ratio (*β* = 1876.81, CI: 194.40 to 3559.21, *p* = 0.0287); and Adenosine 5′-diphosphate (ADP) to glycerol ratio (*β* = 1025.02, CI: 5.31 to 2044.73, *p* = 0.0488). Except for the ratios of dihydroferulic acid sulfate and adenosine 5′-diphosphate (ADP) to glycerol, the MR estimates derived from the MR-Egger regression, weighted mode, weighted median, and simple mode for the remaining metabolites showed consistent directions, supporting the robustness of the causal relationships (Supplementary Table S4; Figure 2).

The heterogeneity test results (MR-Egger and IVW) for blood metabolites in the brain cortex surface area were *p* > 0.05, indicating no heterogeneity. Through the MR Egger test for horizontal pleiotropy, the multiple-effect test results of the blood metabolites with brain cortex surficial area were all *p* > 0.05, indicating no horizontal pleiotropy (Supplementary Table S4). The MR PRESSO test, scatter (Supplementary Figure S1), and funnel plots (Supplementary Figure S2) ruled out potential outliers and horizontal pleiotropy for all identified metabolites. In the “leave-one-out” analysis, each SNP was individually excluded from the analysis (Supplementary Table S5; Supplementary Figure S3). This analysis revealed that no single SNP significantly impacted the robustness of the results. Therefore, the results of the TSMR correlation analyses are stable and reliable. These five metabolite ratios and 11 metabolites were identified as potential candidate metabolites involved in the pathogenesis of epilepsy for further analysis.

### Causal relationship analysis between blood metabolites, ratios, and brain cortex thickness

**Figure 3:**
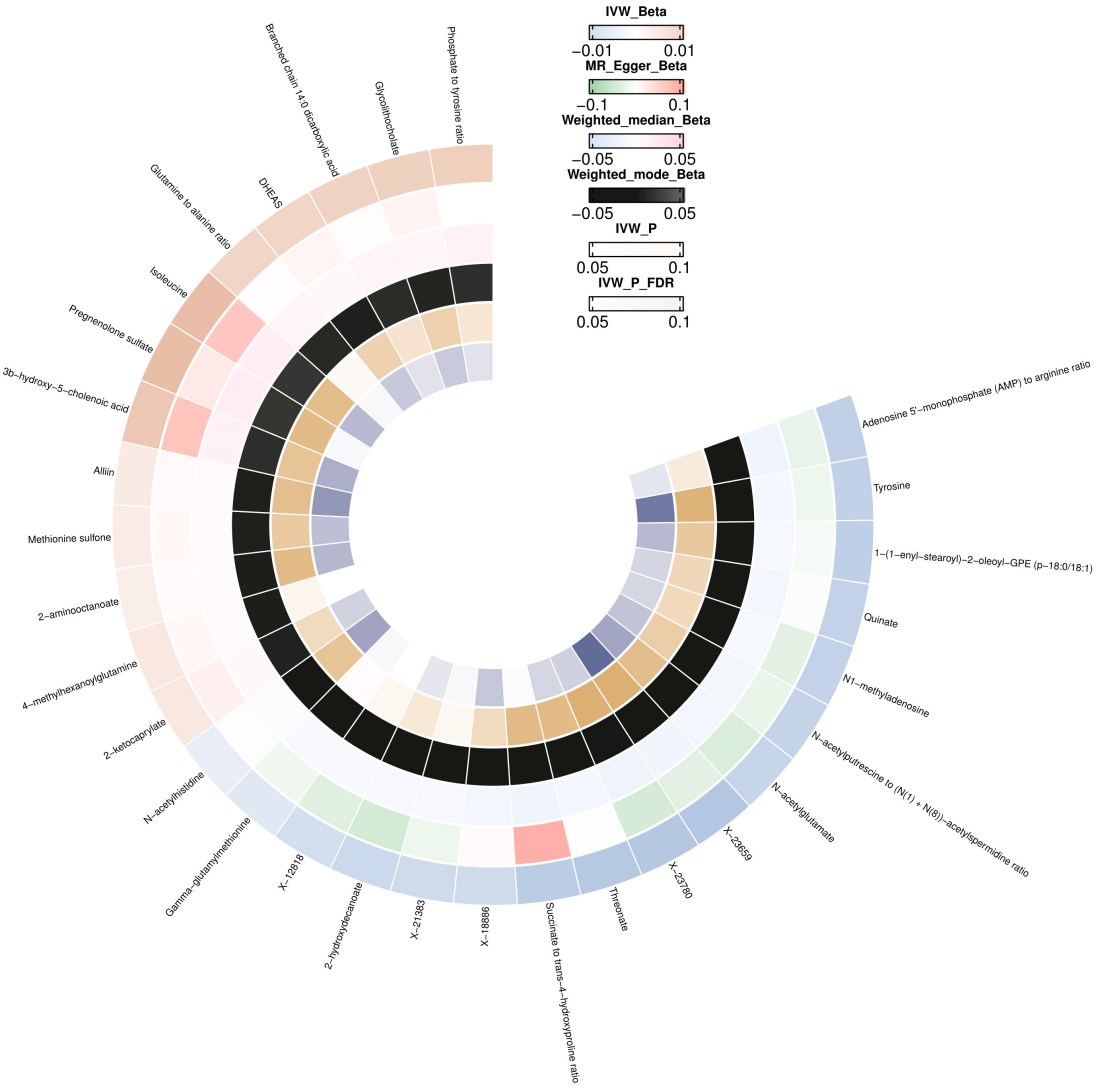
Mendelian randomization associations between blood metabolites and brain cortex thickness.

IVW initially identified 28 causal relationships with multiple testing correction significance (FDR <0.05). In addition to the five metabolites with unknown chemical compositions, another 18 metabolites and five metabolite ratios were categorized as amino acids, lipids, peptides, xenobiotics, energy, nucleotides, cofactors, and vitamins. By conducting a sensitivity analysis, all of these met the criteria for eligible candidate metabolites affecting the brain cortex thickness, including Isoleucine (*β* = 0.0172, CI: 0.0041 to 0.0304, *p* = 0.0101), Tyrosine (*β* = −0.0156, CI: −0.0258 to −0.0054, *p* = 0.0025), Branched chain 14:0 dicarboxylic acid (*β* = 0.0118, CI: 0.0009 to 0.0226, *p* = 0.0331), Pregnenolone sulfate (*β* = 0.0171, CI: 0.0044 to 0.0299, *p* = 0.0081), Methionine sulfone (*β* = 0.0048, CI: 0.0008 to 0.0087, *p* = 0.0389), DHEAS (*β* = 0.0110, CI: 0.0015 to 0.0205, *p* = 0.0230), Threonate (*β* = −0.0183, CI: −0.0319 to −0.0046, *p* = 0.0430), Glycolithocholate (*β* = 0.0121, CI: 0.0017 to 0.0226, *p* = 0.0408), N-acetylglutamate (*β* = −0.0142, CI: −0.0251 to −0.0033, *p* = 0.0102), Quinate (*β* = 0.0152, CI: −0.0286 to −0.0018, *p* = 0.0256), 3b-hydroxy-5-cholenoic acid (*β* = 0.0147, CI: 0.0030 to 0.0264, *p* = 0.0132), 2-hydroxydecanoate (*β* = −0.0131, CI: −0.0254 to −0.0008, *p* = 0.0366), 2-aminooctanoate (*β* = 0.0041, CI: 0.0010 to 0.0072, *p* = 0.0087), Alliin (*β* = 0.0050, CI: 0.0011 to 0.0088, *p* = 0.0105), 1-(1-enyl-stearoyl)-2-oleoyl-GPE (p-18:0/18:1) (*β* = −0.0161, CI: −0.0292 to −0.0029, *p* = 0.0163), Gamma-glutamylmethionine (*β* = −0.0079, CI: −0.0158 to −3.2811E-05, *p* = 0.0490), N1-methyladenosine (*β* = −0.0152, CI: −0.0287 to −0.0017, *p* = 0.0430), N-acetylhistidine (*β* = −0.0056, CI: −0.0100 to −0.0011, *p* = 0.0135), 2-ketocaprylate (*β* = 0.0061, CI: 0.0006 to 0.0115, *p* = 0.0281), Glutamine to alanine ratio (*β* = 0.0109, CI: 0.0001 to 0.0216, *p* = 0.0472), N-acetylputrescine to N(1) + N (8)-acetylspermidine ratio (*β* = −0.0146, CI: −0.0270 to −0.0023, *p* = 0.0198), Adenosine 5′-monophosphate (AMP) to arginine ratio (*β* = −0.0157, CI: −0.0306 to −0.0008, *p* = 0.0380), Phosphate to tyrosine ratio (*β* = 0.0125, CI: 0.0009 to 0.0241, *p* = 0.0345) (Supplementary Table S6; Figure 3).

We did not observe horizontal pleiotropy or heterogeneity using the MR-Egger intercept or Cochran’s *Q* test (Supplementary Table S6). No outliers were detected using the MR PRESSO test (Supplementary Table S6), leave-one-out analysis (Supplementary Table S7; Supplementary Figure S4), or scatter and funnel plots (Supplementary Figures S5, S6).

### Causal analysis of brain cortex thickness on blood metabolites

**Figure 4:**
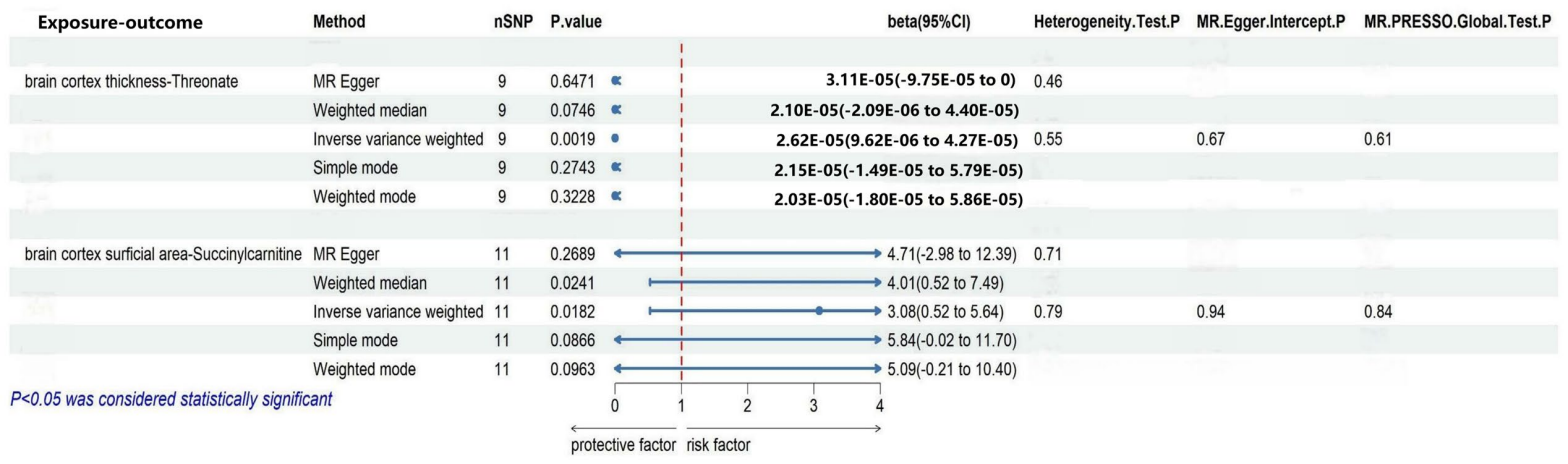
Sensitivity analysis of significant casual associations in blood metabolites.

We selected nine eligible SNPs from a large-scale European brain cortex thickness genome-wide association study (GWAS). Our reverse analysis showed a significant causal effect of improved cerebral cortex thickness leading to elevated threonate levels (*β*_IVW_ = 3.0799, CI: 0.5235 to 5.6363, *p* = 0.0182). Estimates using the MR Egger approach (*β*_IVW_ = 4.7052, CI: −2.9767 to 12.3871, *p* = 0.2689) and weighted median method (*β*_IVW_ = 4.0082, CI: 0.5234 to 7.4930, *p* = 0.0241) showed similar trends. The MR PRESSO analysis revealed no outlying SNPs. Based on the leave-one-out analyses, no single SNP affected the causative estimate of the brain cortex thickness for blood metabolites (Figure 4).

### Causal analysis of brain cortex surficial area on blood metabolites

To explore the causal effect of the brain cortex surface area as an exposure factor for blood metabolites, 11 SNPs were selected using the method described above. The IVW analysis showed a trend-causing relationship between the brain cortex surficial area and succinylcarnitine levels (*β* = 2.62 × 10^−05^, CI: 9.62 × 10^−06^ to 4.24 × 10^−05^, *p* = 0.0019). Estimates based on the MR Egger (*β* = 3.11 × 10^−05^, CI: −9.75 × 10^−05^ to 0.0001, *p* = 0.6470) and weighted median (*β* = 2.10 × 10^−05^, CI: −2.09 × 10^−06^ to 4.40 × 10^−05^, *p* = 0.0746) show a similar trend. Furthermore, the heterogeneity and horizontal pleiotropy test results of the brain cortex surface area with succinylcarnitine levels were *p* > 0.05, suggesting no heterogeneity or horizontal pleiotropy; therefore, the results are not biased. No outlying SNPs were found using the MR PRESSO analysis. Finally, the leave-one-out analysis validated our results (Figure 4).

### Metabolic pathway analysis

**Figure 5:**
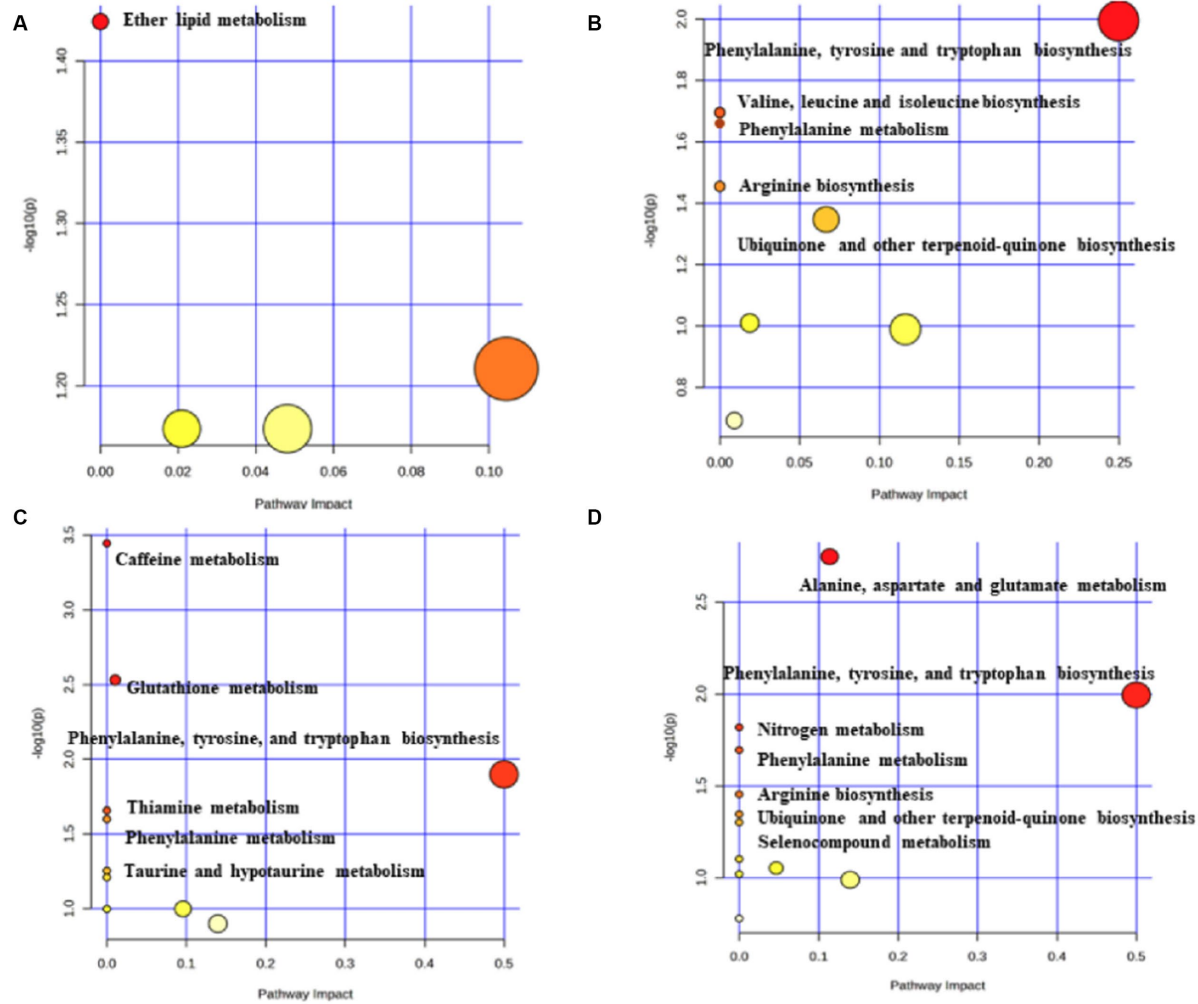
Potential metabolic pathways associated with brain cortex surficial area and cortex thickness. **(A)** Potential metabolic pathways involved in cortex surficial area alterations in MR positive analysis (based on metabolites). **(B)** Potential metabolic pathways involved in cortex thickness alterations in MR positive analysis (based on metabolites). **(C)** Potential metabolic pathways involved in cortex surficial area alterations in MR positive analysis (based on metabolite ratios). **(D)** Potential metabolic pathways involved in cortex thickness alterations in MR positive analysis (based on metabolite ratios). Pathway analysis based on “Kyoto Encyclopedia of Genes and Genomes” (KEGG). The color and size of each circle is based on *p*-values (yellow: higher *p*-values and red: lower *p*-values) and pathway impact values (the larger the circle the higher the impact score) calculated from the topological analysis, respectively.

The metabolic pathway analysis based on metabolites identified six significant metabolic pathways in the cortical surface area and cortical thickness (Supplementary Table S8). Our results showed that the “ether lipid metabolism” (*p* = 0.0376) was found to be associated with the improvement in the cortex surficial area (Figure 5A). There are five metabolic pathways associated with cortex thickness, with the most significant pathway being the “phenylalanine, tyrosine, and tryptophan biosynthesis” (*p* = 0.01013) (Figure 5B).

The metabolic pathway analysis based on the metabolite ratios identified 13 important metabolic pathways associated with the cortical surface area and thickness (Supplementary Table S9). Among the six metabolic pathways related to the cortex surficial area, the most notable is the “caffeine metabolism” (*p* = 3.59×10^−4^) (Figure 5C). In the case of cortex thickness, among the seven metabolic pathways, the most significant is the “alanine, aspartate, and glutamate metabolism” (*p* = 0.0017) (Figure 5D).

## Discussion

**Figure 6:**
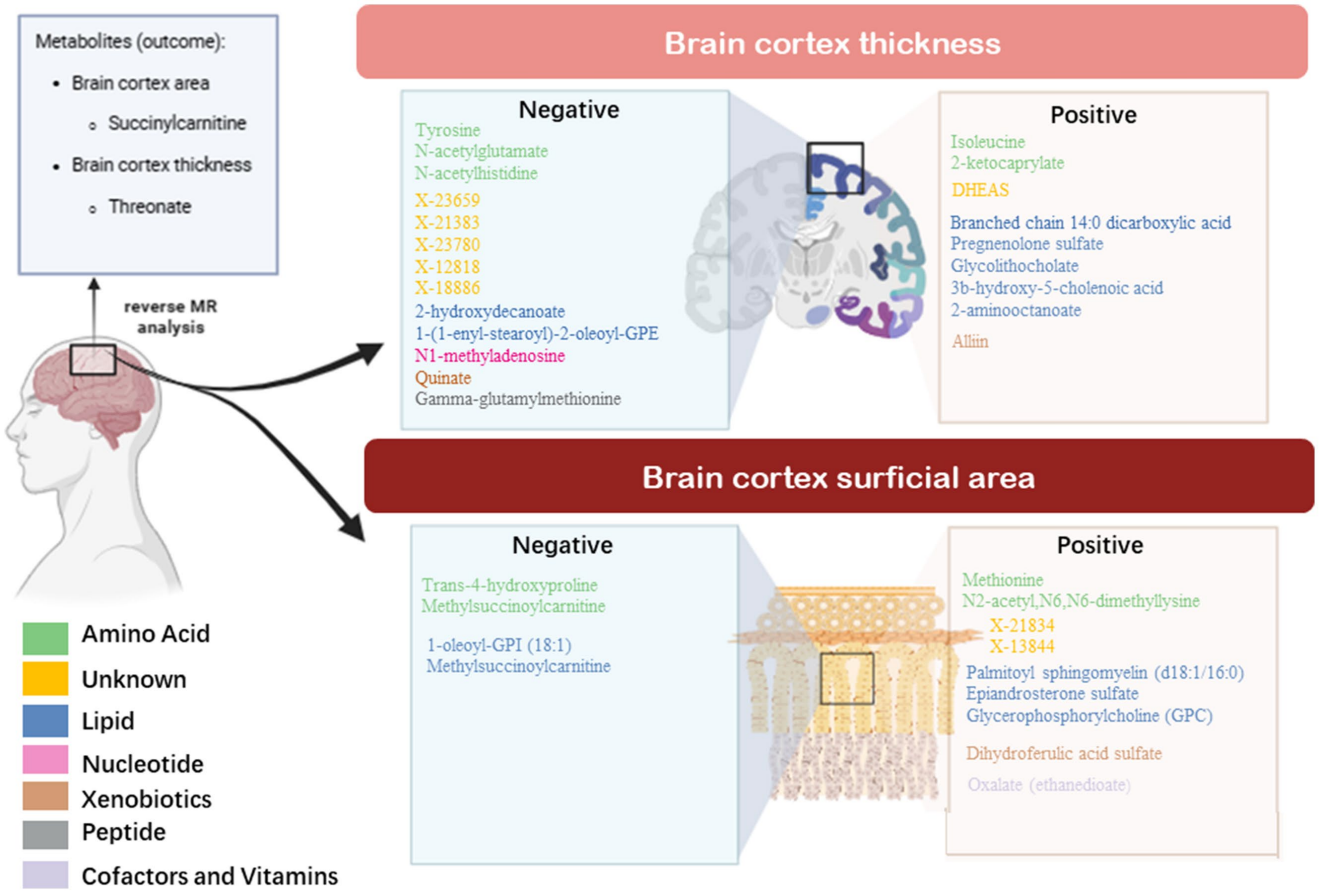
Bidirectional casual links between metabolites and cortex architecture.

In this two-sample MR study, we found 57 causal relationships involving 45 metabolites and 12 metabolite ratios, 36 of which were observed with multiple-testing-corrected significance and were considered as robust associations (Figure 6). In addition, 19 metabolic pathways associated with brain cortex thickness and surface area were identified. To our knowledge, this was the first MR study to apply the most comprehensive blood metabolite GWAS data to explore the causality with the brain cortical structure and to incorporate the metabolic pathways and reverse MR analysis. These findings offer new insights into the impact of metabolites on brain health and offer potential for further precision treatments in the context of neurodegenerative diseases.

### Effect of blood metabolites on the brain cortex surface area

We identified four metabolites from the amino acid metabolic pathways that have been causally associated with pathogenic processes in the brain cortex surface area. Amino acids are substrates for the synthesis of proteins and other small molecule compounds (dopamine, glutathione, nitric oxide, etc.) (14). Studies have shown that amino acids are valuable for regulating nutritional, immune, and oxidative stress and are important for the development of the cerebral cortex in both humans and animals (15). Among these amino acid-related metabolites, methionine and trans-4-hydroxyproline have received considerable attention.

The high-energy requirements and vulnerability of the brain to oxidative stress result in developmental processes that are susceptible to mitochondrial damage (16). Methionine is an essential aliphatic sulfur-containing amino acid. Its breakdown produces glutathione (GSH), taurine, and other metabolites that interfere with lipid metabolism and activate endogenous antioxidant enzymes that combat oxidative stress. Therefore, it protects the cerebral cortex from mitochondrial damage and oxidative stress (17). Mahmarzayeva et al. (18) revealed that methionine metabolites could protect against galactosamine-induced encephalotoxicity by reversing the antioxidant enzyme content and antioxidant status levels. In addition, existing research has confirmed that heavy metal deposits, such as Pb and Ni, can lead to a reduction in the volume of the cerebral cortex. Methionine can chelate and remove lead and nickel from the brain tissue, thereby reducing oxidative stress and protecting the brain structure (6, 19). These studies suggest that rational methionine supplementation may interfere with the antioxidant capacity of an organism, thereby reducing DNA damage, protecting the brain structure, and delaying neurodegenerative diseases. Our results further support these findings and highlight the value of methionine as a protective factor for the brain cortex surface area.

Trans-4-hydroxyproline, one of the hydroxyproline (Hyp) isomers, is an abundant component of mammalian collagen that functions as a chiral synthon in the synthesis of anti-inflammatory drugs. A targeted metabolomic approach revealed higher levels of trans-4-hydroxyproline in the cerebrospinal fluid of patients with Parkinson’s disease (PD) than those in the control subjects (20). We hypothesized that elevated levels of trans-4-hydroxyproline in the body fluids of patients with PD may be related to neurodegeneration and increased collagen degradation. Trans-4-hydroxyproline was found to be an important substrate for conotoxin constitution (21). Conotoxins are novel neurotoxins that block or inhibit voltage-gated sodium channels in the central nervous system (CNS). It functions in the development of cortical structures at several levels, including neurite outgrowth, axonal pathfinding, dendritic arborization, neuronal patterning, and cell migration (22). Based on the evidence provided and our results, trans-4-hydroxyproline can be considered an important amino acid biomarker and key therapeutic target for neurodegenerative diseases.

In addition to the amino acid metabolism pathway, we identified five metabolites from the lipid metabolism pathways. Among these metabolites, epiandrosterone sulfate and glycerophosphorylcholine (GPC) have received considerable attention. Epiandrosterone sulfate is associated with androgen metabolism. Previous studies have shown that androgen depletion can increase the susceptibility to neurodegenerative diseases (23). In addition, appropriate supplementation with androgens may be neuroprotective by preventing β-amyloid deposition and the hyperphosphorylation of tau proteins (24). However, the association between genetically determined androgenic steroids (e.g., epiandrosterone sulfate) and neurodegenerative diseases remains obscure. Our study, based on the brain cortex structure GWAS data from 51,665 European participants, found that a higher level of epiandrosterone sulfate was a protective factor in the brain cortex. Thus, it holds promise as a potential therapeutic target for neurodegenerative diseases since it slows down the disease progression.

GPC is a common precursor of choline and acetylcholine in the brain. After oral administration, GPC can cross the blood-brain barrier and reach the CNS. GPC maintains the neuronal integrity by crossing the blood-brain barrier and penetrating the phospholipid portion of the neuronal plasma membrane (25). In an *in vivo* study on oxidative stress, Yamanaka et al. (26) found that GPC metabolites are potent scavengers of reactive oxygen species and prevent lipid peroxidation. Brownawell et al. (27) found that GPC can be converted to PC to support the mitochondria or increase the production of acetylcholine (Ach) in the brain, thereby affecting the brain cortex development. Our results are consistent with previous findings and further validate that GPC could be a drug target for brain protection.

Dihydroferulic acid sulfate is a ferulic acid derivative used in xenobiotic pathways. Although studies on dihydroferulic acid sulfate in the nervous system are rare, Serreli et al. (28) showed that it reduces the degradation of nitric oxide by reducing superoxide formation. It was also found to counteract the inflammatory response of different signaling pathways and maintain intestinal integrity (29). Therefore, we speculated that the protective effect of dihydroferulic acid sulfate on the cortex is mediated by its anti-inflammatory and antioxidant activities. We have also detected oxalic acid from the pathways of cofactors and vitamins, which have a protective effect on cortical regions of the brain. High concentrations of oxalic acid can chelate iron, reducing the production of free radicals and inhibiting lipid peroxidation. An *in vitro* study found that oxalic acid prevents lipid peroxidation in rat liver mitochondria and brain in a concentration-dependent manner. Considering the significant differences between animal and human studies, establishing a strong connection between animal and human research is crucial (29, 30). Therefore, future research should be guided by previous studies to further confirm the antioxidant mechanisms of oxalic acid in human studies. Given the importance of oxidative stress in brain health, dihydroferulic acid sulfate and oxalate may be promising targets for further studies.

### Effect of metabolite ratios on the brain cortex surface area

This causality was not significantly associated with either of the two metabolites forming the ratio, suggesting that forming the ratio could help identify a significant causal relationship by focusing on specific metabolic reaction pathways. In this study, we identified the metabolic pathways that were causally associated with the development of the cortical area by identifying the relevant metabolite ratios. The role of some pathways in the development of the cortex has been well-documented in experimental studies.

Caffeine metabolism reaction alters the functional brain connectivity and regulates brain development by altering the synaptic and metabolic processes (31). Lopes et al. (32) found that caffeine metabolism increases the metabolic capacity of synapses by altering the energy charge and redox state of cortical synapses, thus helping the cortex respond to noxious stimuli. Among these metabolic reactions, the altered functional status of thiamine metabolism has been characterized as a major pathogenic mechanism in Alzheimer’s disease (33). Using *in vivo* and *in vitro* mammalian assays demonstrated that the regulation of pyridoxal kinase in thiamine metabolism can affect brain homeostasis (34). Taurine and hypotaurine have antioxidative, osmoregulatory, and anti-inflammatory functions (35). Bousquet et al. (36) confirmed that taurine and hypotaurine metabolic pathways have important clinical implications in neurodegenerative diseases by studying key cystamine metabolites, including cysteamine, hypotaurine, taurine, and cysteine.

### Effect of blood metabolites on the brain cortex thickness

Our research indicates that multiple metabolites affect the cortex thickness and that the specific mechanisms may be diverse. There were six metabolites in the amino acid metabolism pathway. Tyrosine serves as a precursor of dopamine and norepinephrine, and its effects on brain tissue have been controversial in recent years (37). Oxidative stress and metabolic disorders in the brain due to tyrosine damage to the mitochondria can be recapitulated using *in vivo* and *in vitro* experimental models (38). However, a longitudinal study found that tyrosine can exert notable acute cognitive and emotional benefits in some individuals (39). Based on 51,665 European individuals, the present MR study found a negative association between tyrosine levels and the risk of cortex thickness. Like tyrosine, N-acetylglutamate disrupts mitochondria by decreasing the mitochondrial membrane potential and inducing Ca^2+^ overload, thereby affecting brain development (40). Another finding of this study noted that N1-methyladenosine from the nucleotide pathways acts as a risk factor for cortical thickness. Previous studies have revealed a significant elevation in N1-methyladenosine levels in the plasma of patients with stroke. This elevation was associated with the degree of brain damage (41). In this study, genetically determined higher N1-methyladenosine levels correlated with a higher risk of cortical structure destruction, supporting the previously mentioned conclusion.

Pregnenolone sulfate (PREGS) from the lipid pathway exerts protective effects on cortical thickness. PREGS is a controversial neurosteroid (42). It increases neuronal activity by inhibiting GABAergic activity and stimulating glutamatergic neurotransmission (43). PREGS-mediated increase in neuronal excitability may be dangerous under specific conditions, for example in the case of excitotoxic (44). However, the physiological significance of these observations has recently been questioned due to the failure to detect significant levels of PREGS in the brain and plasma of mice (44). Given that a protective effect was identified by MR analysis, we believe that PREGS could serve as a novel and promising drug target.

Alliin, the most abundant sulfur compound in garlic, has been demonstrated to exhibit hypoglycemic, antioxidant, and anti-inflammatory properties. In the present study, alliin acted as a protective factor against cortical thickness, which is consistent with the findings of previous studies (45).

### Effect of metabolite ratios on brain cortex thickness

Our study identified meaningful metabolic pathways by determining metabolite ratios and the enrichment of specific metabolites. Several of these pathways have been extensively documented in animal models and in plasma studies. Among the seven metabolic pathways related to cortical thickness, the most notable were the alanine, aspartate, and glutamate metabolic pathways. This metabolic pathway affects aspartate, glutamate, and γ-aminobutyric acid (GABA) levels, which play key roles in early brain development (46). In addition, the involvement of the arginine biosynthesis and aspartate and glutamate metabolism has been identified in studies involving epilepsy, schizophrenia, AD animal models, and blood samples (47–50). Our study provides a stronger validation of these conclusions.

Phenylalanine, tyrosine, and tryptophan biosynthesis and phenylalanine metabolism are closely associated with the etiology of several psychiatric disorders (51). The products of the reaction affect catecholamine synthesis, neurotransmitter release, and the connections between neurons (52). Thus, in conjunction with the present study, the clinical significance of phenylalanine, tyrosine, and tryptophan biosynthesis has been suggested from a genetic perspective. Additionally, studies of the correlation of selenocompound metabolism and nitrogen metabolism with the CNS are rare, but metabolites during the reactions of both pathways can modulate the redox state, affect mitochondrial function, and influence brain development (53, 54). Moreover, we discovered the involvement of ubiquinone and other terpenoid-quinone biosynthesis pathways in cortical thickness. However, the specific mechanisms underlying these findings require further investigation.

In this study, we also identified the metabolic pathways causally linked to cortical structures based on relevant metabolites. Some of these have been well-documented in experimental studies on the development of the cortex. As mentioned above, phenylalanine, tyrosine, tryptophan biosynthesis, arginine biosynthesis, and phenylalanine metabolism are involved in the cortical structure. Valine, leucine, and isoleucine are involved in energy metabolism and biosynthesis and play a role in regulating brain metabolism and neurotransmitter synthesis (55). Additionally, Teigler et al. (56) found that impaired ether lipid metabolism leads to structural defects in the cerebellum and peripheral myelin formation. However, the role of ether lipid metabolism in mammals and the underlying molecular mechanisms remain largely unknown.

Our study had several advantages. To the best of our knowledge, this was the first comprehensive MR study based on genomic and metabolomic data to identify novel risk factors for cortical development. Second, this MR analysis had an extensive coverage of genetic variables, which enabled us to make valid causal inferences. Finally, bidirectional MR designs largely avoid reverse causation and residual confounders (57).

However, this study had several limitations. First, this was a blood metabolome-based analysis. Therefore, our study framework could be extended to include additional metabolites. Second, the study included participants of European ancestry. Future studies using GWAS data from other races are needed to test the generalizability across populations. Third, some metabolites and metabolic pathways covered in this study have not been fully elucidated concerning their functions and mechanisms in cortical development, which limits our interpretation of the MR analysis results.

## Conclusion

This two-sample MR study revealed the significant role of serum metabolites in cortex development and health. We identified significant causal associations between 37 metabolites and nine metabolite ratios with cortical thickness and surface area and identified 17 significant metabolic pathways based on these metabolites and ratios. These results reveal the importance of targeting metabolites to protect cortical health and identify potential therapeutic pathways to reduce the risk of neurodegenerative diseases. Ultimately, these findings will help guide health policies and clinical practices in treating neurodegenerative diseases in the context of population aging.

## Data availability statement

The original contributions presented in the study are included in the article/Supplementary material, further inquiries can be directed to the corresponding author.

## Ethics statement

Ethical approval was not required for the study involving humans in accordance with the local legislation and institutional requirements. Written informed consent to participate in this study was not required from the participants or the participants’ legal guardians/next of kin in accordance with the national legislation and the institutional requirements.

## Author contributions

ZJ: Data curation, Investigation, Methodology, Project administration, Writing – original draft. YS: Formal analysis, Project administration, Supervision, Writing – original draft, Writing – review & editing. SL: Funding acquisition, Resources, Validation, Writing – review & editing.

## Footnotes

^1^http://www.ebi.ac.uk/gwas/

^2^http://mrcieu.github.io/TwoSampleMR/

^3^https://www.metaboanalyst.ca
