# Supplementary material for: Association between human blood metabolites and cerebral cortex architecture: evidence from a Mendelian randomization study
Source: Front Neurol. 2024 May 9;15:1386844. doi: 10.3389/fneur.2024.1386844 (PMC11111910; doi:10.3389/fneur.2024.1386844)

**Fig S1**. Scatter plots from genetically predicted blood metabolites on brain cortex surficial area.


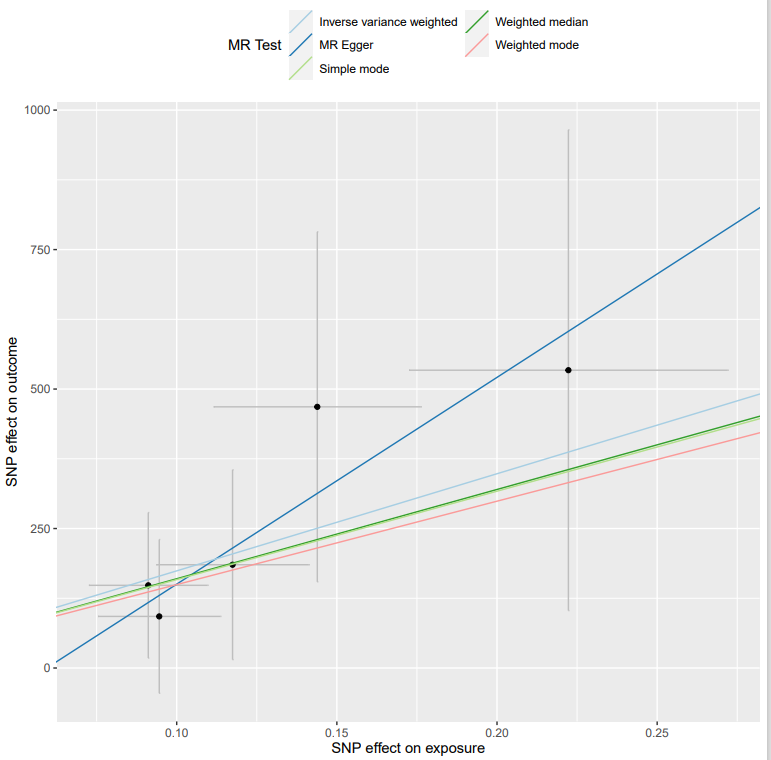

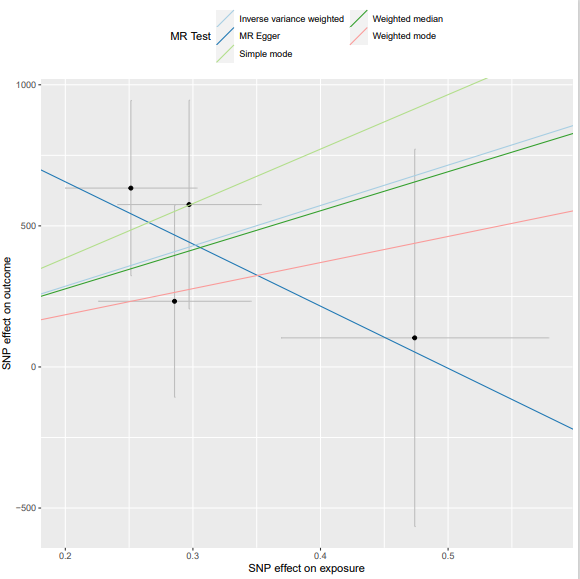


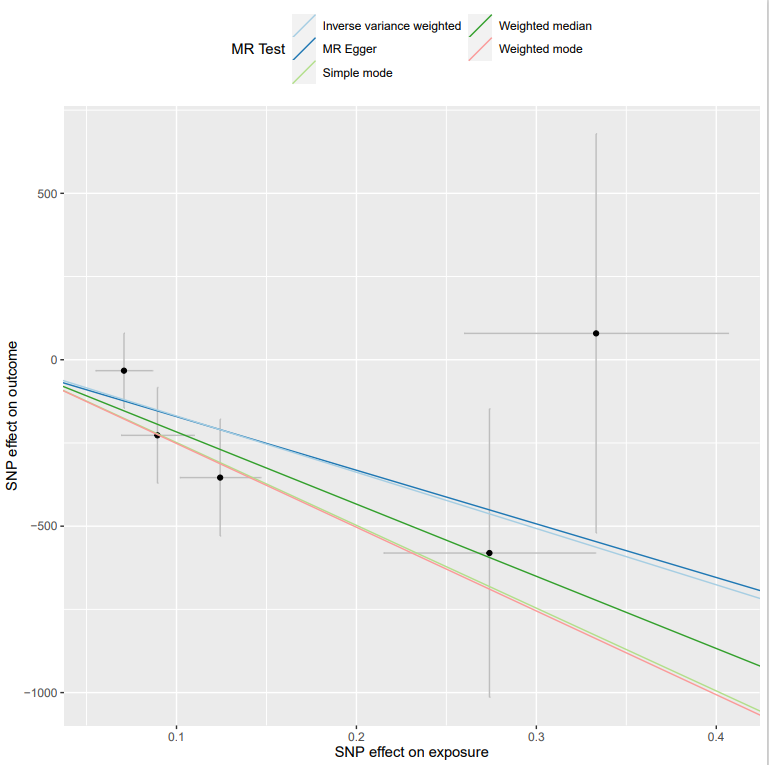

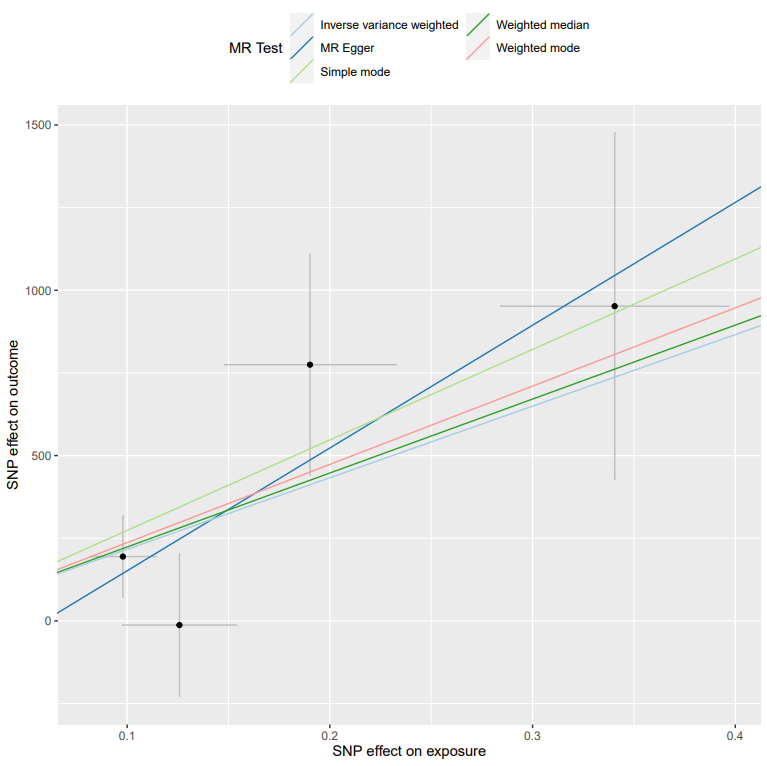


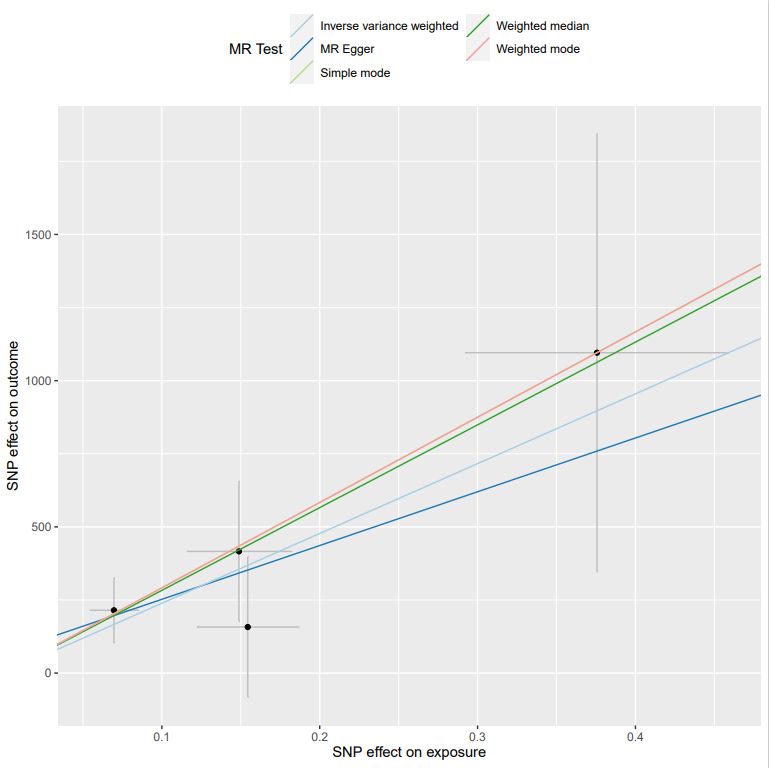

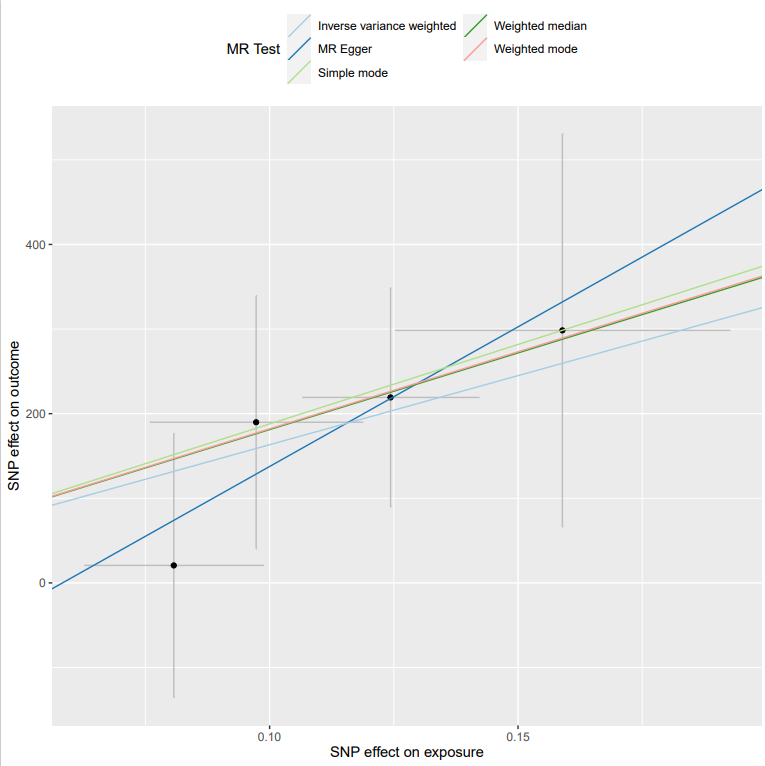


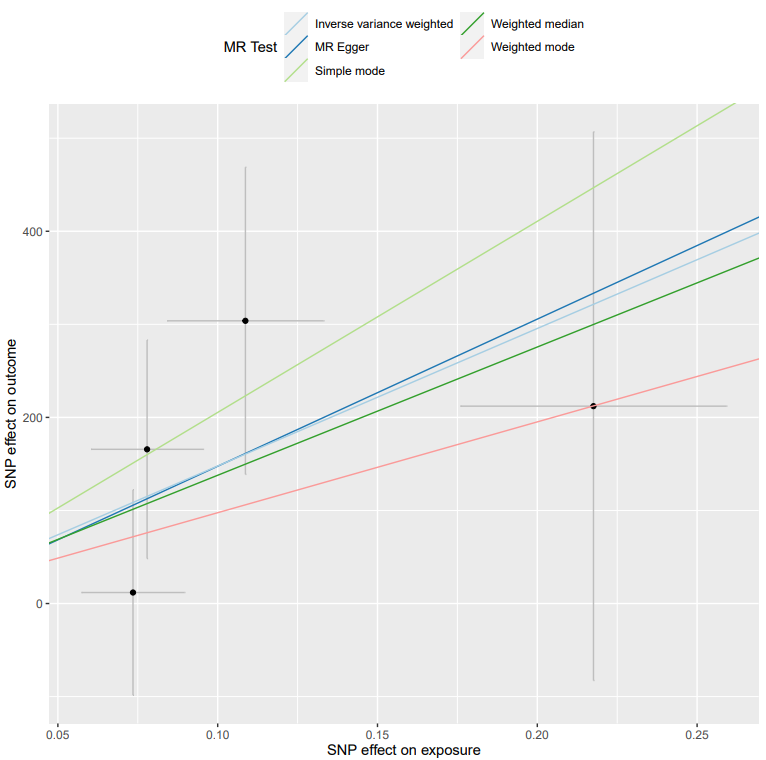

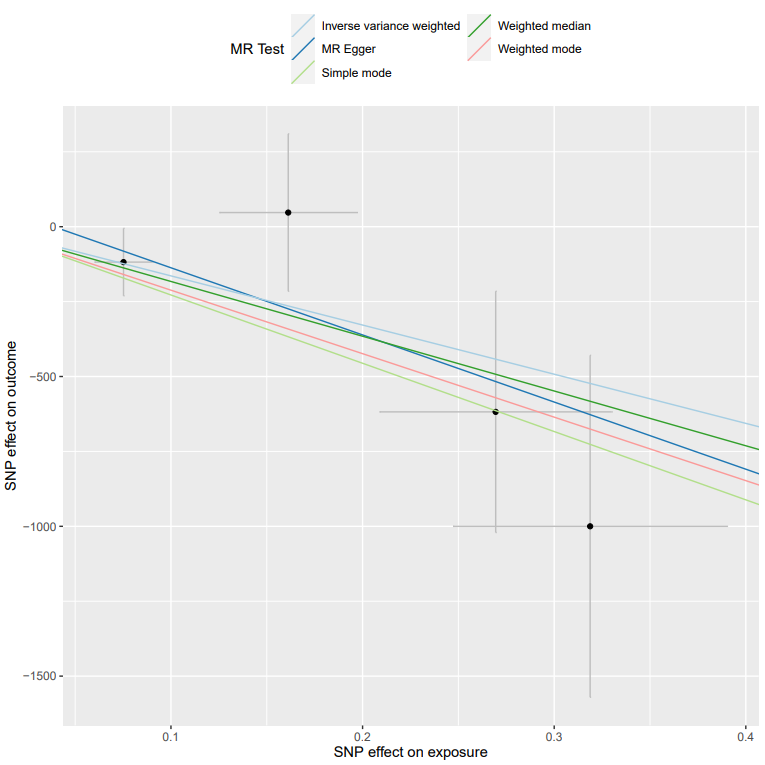


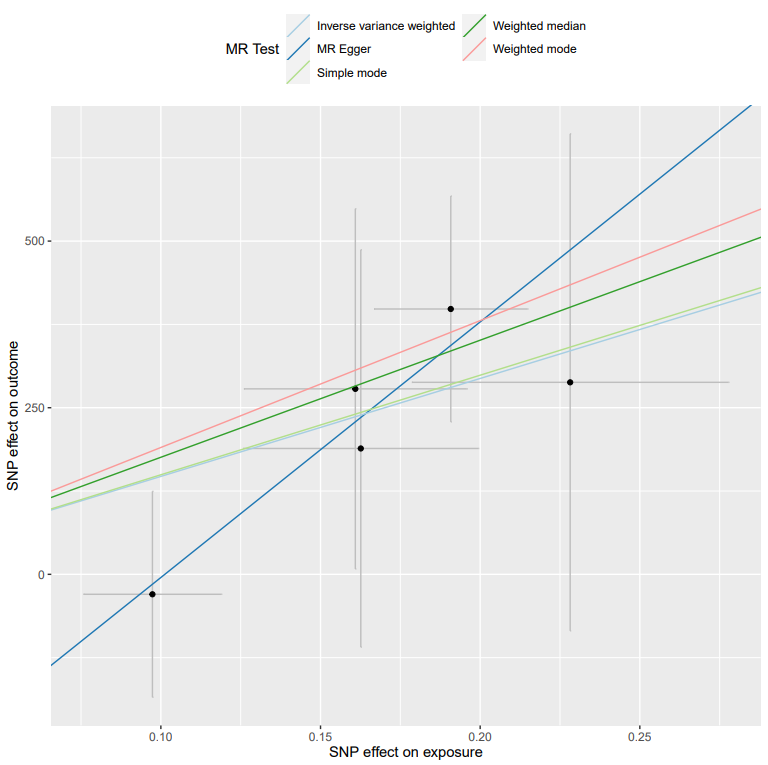

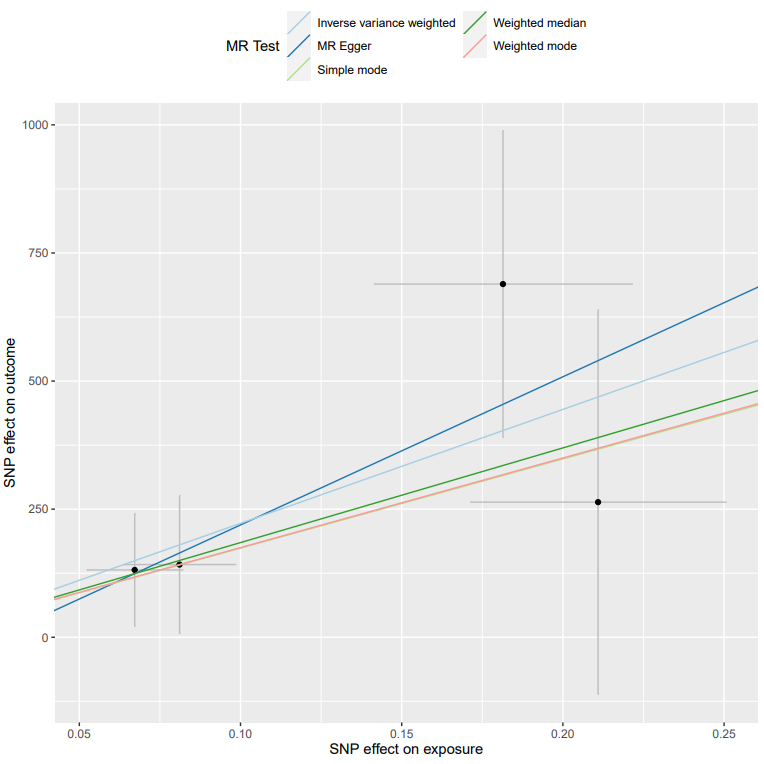


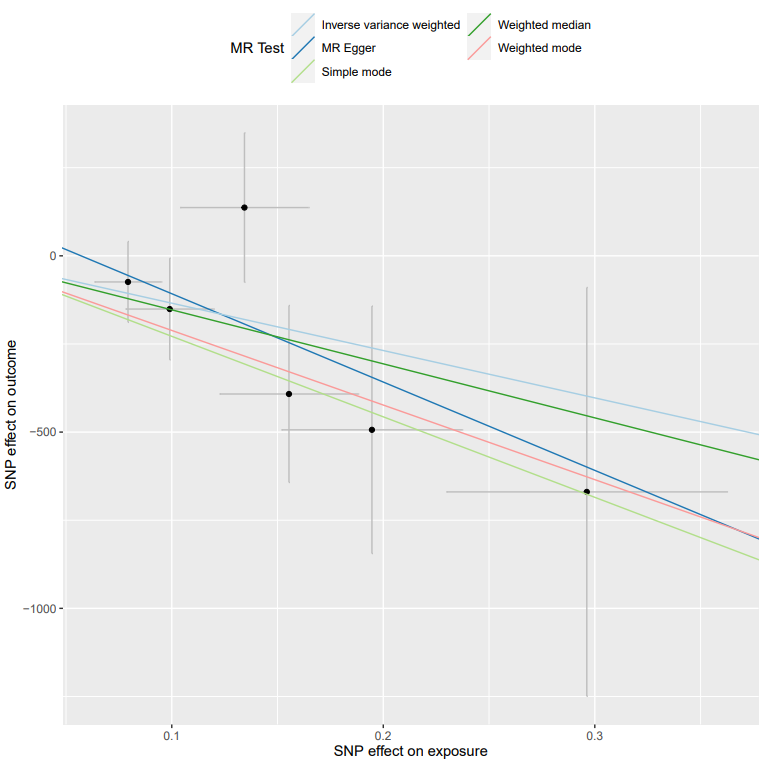

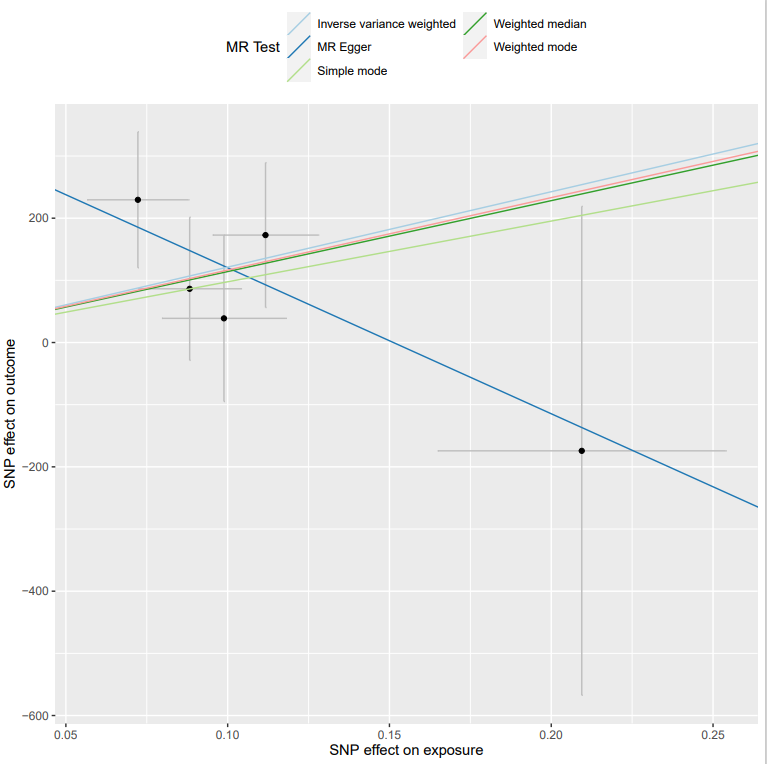


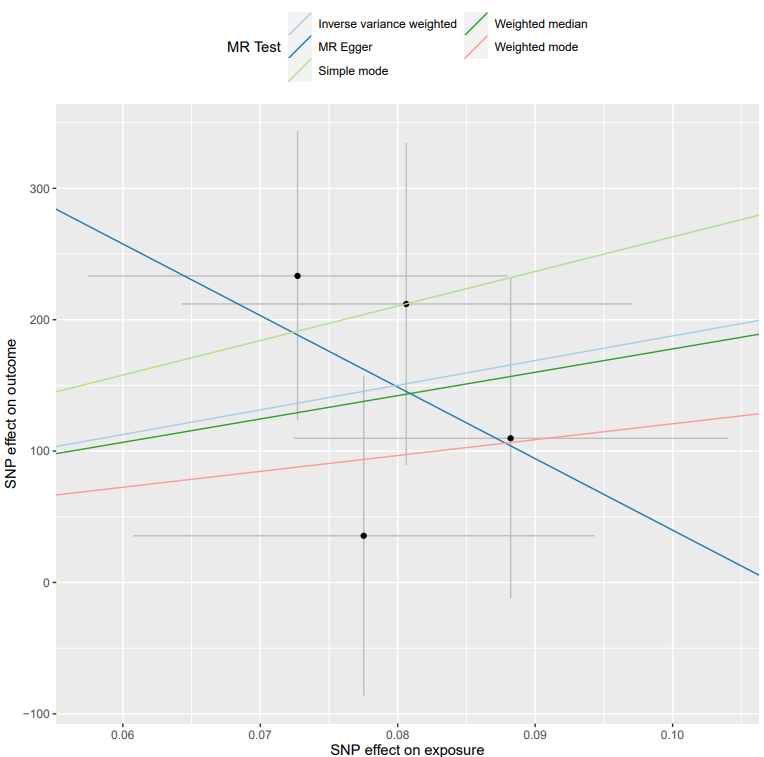

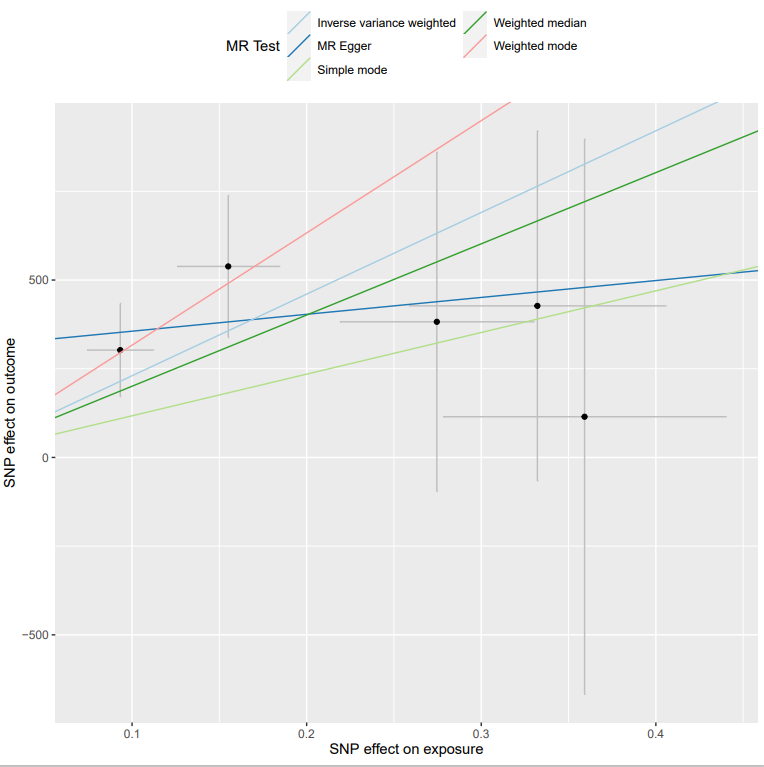


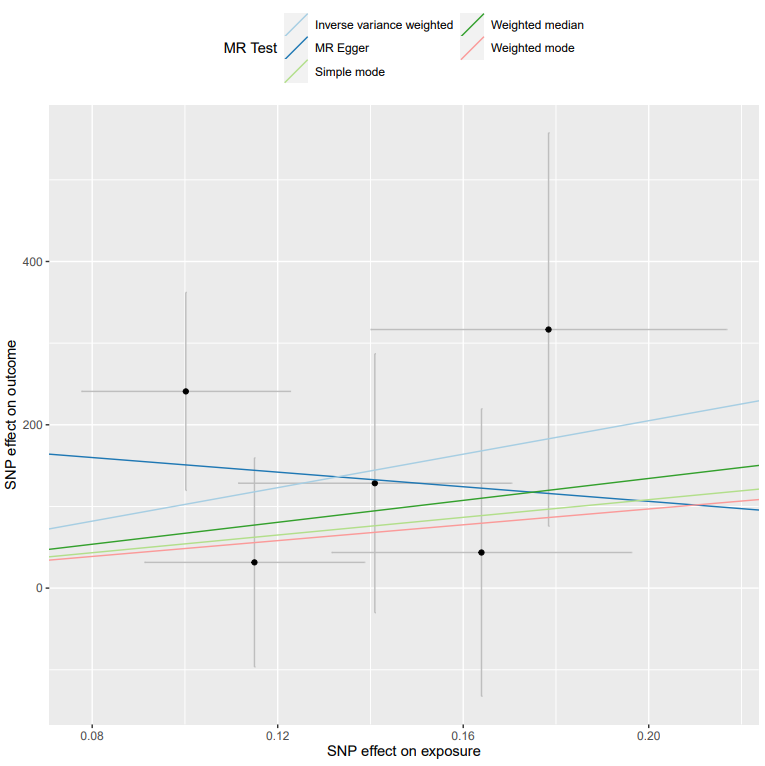


**Fig S2**. Funnel plot from genetically predicted blood metabolites on brain cortex surficial area.


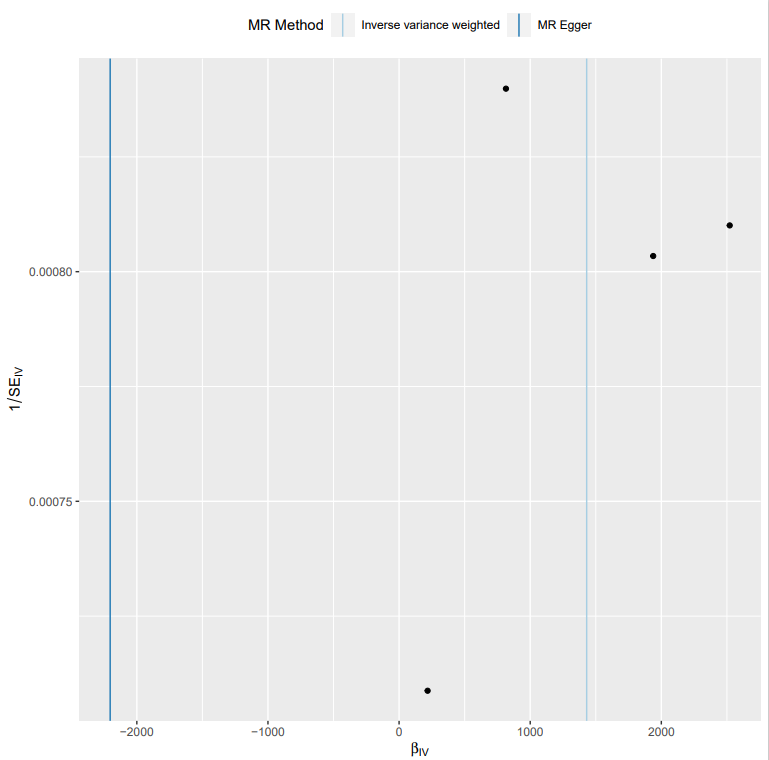

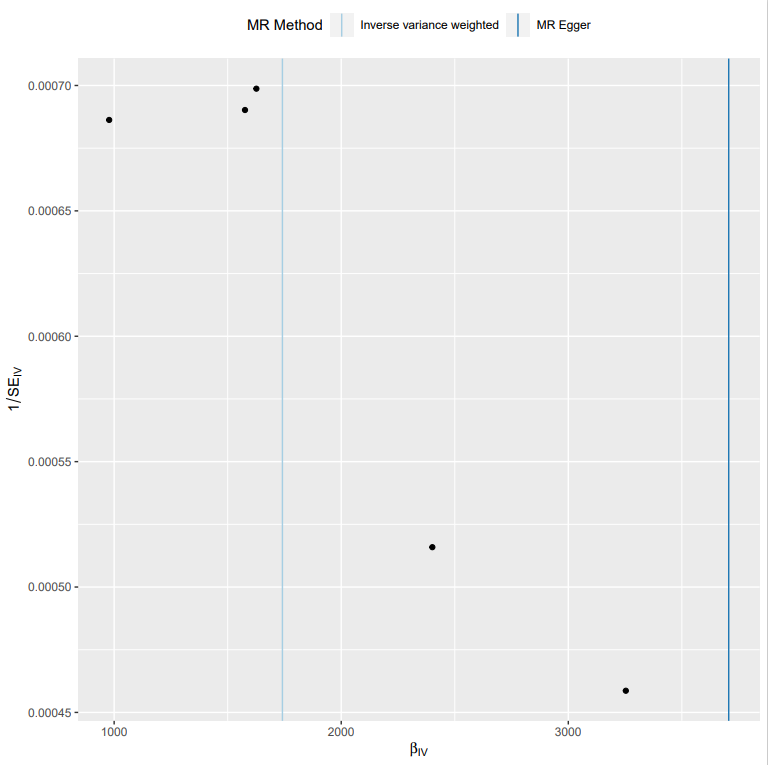


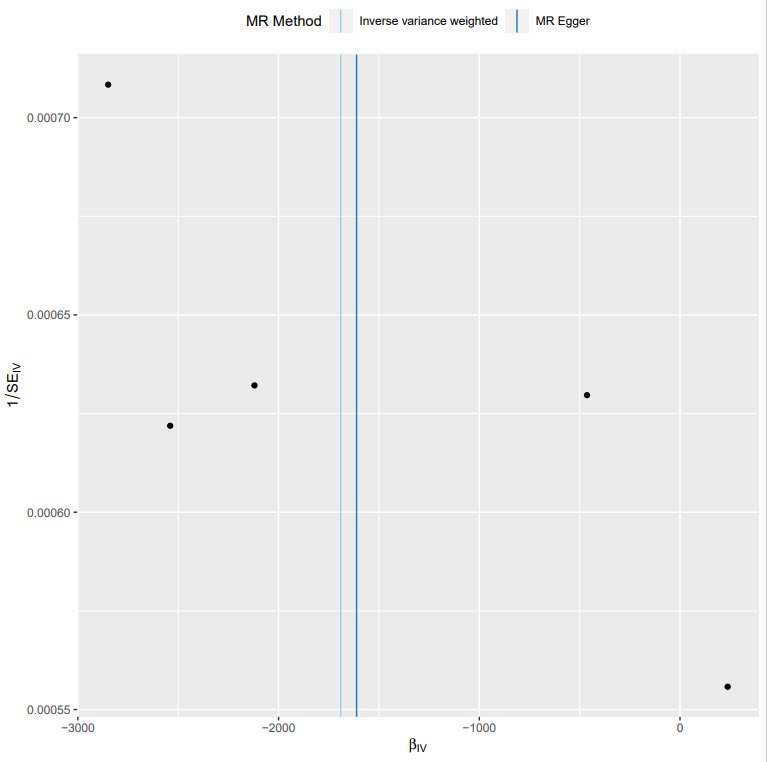

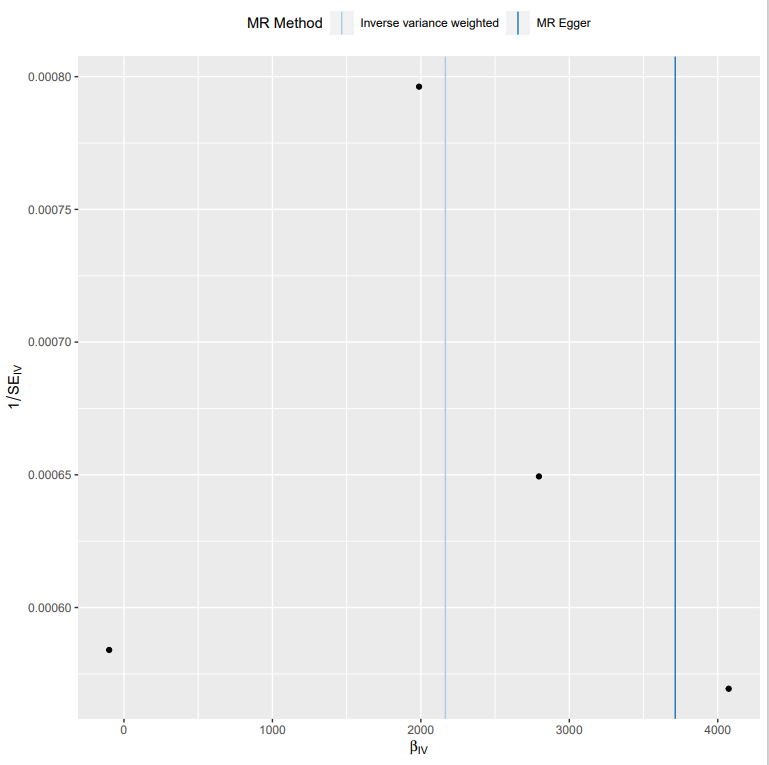


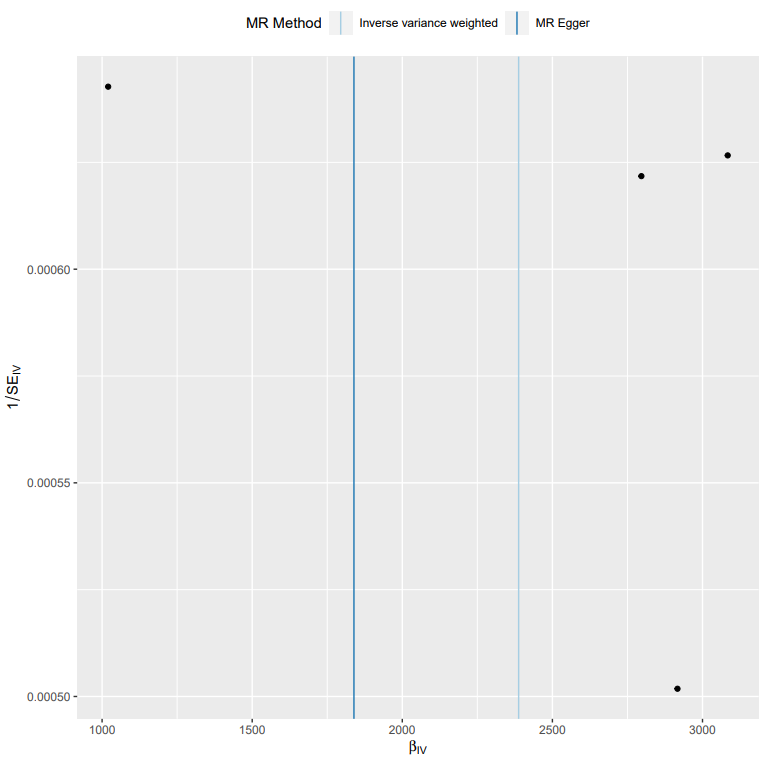

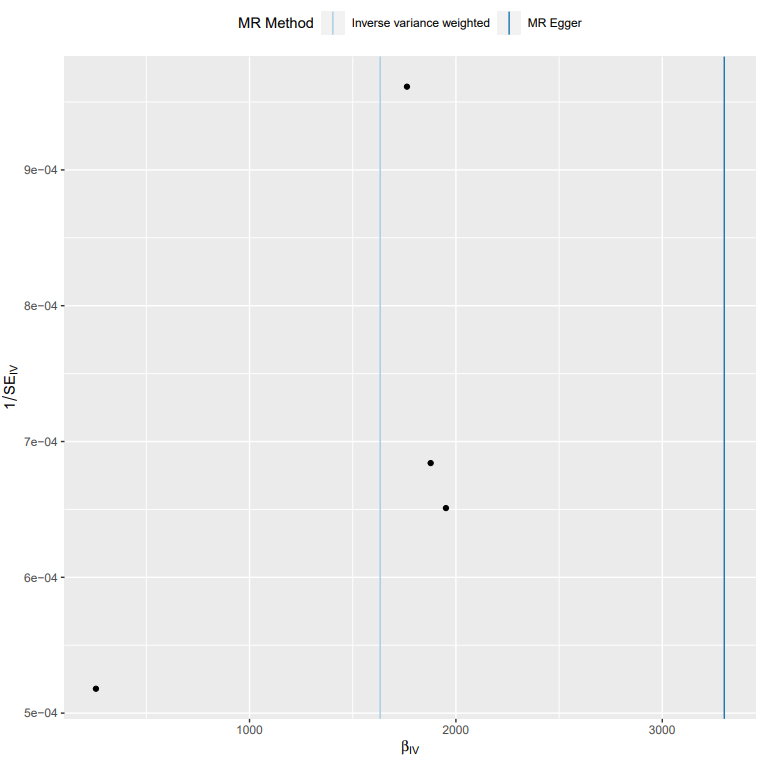


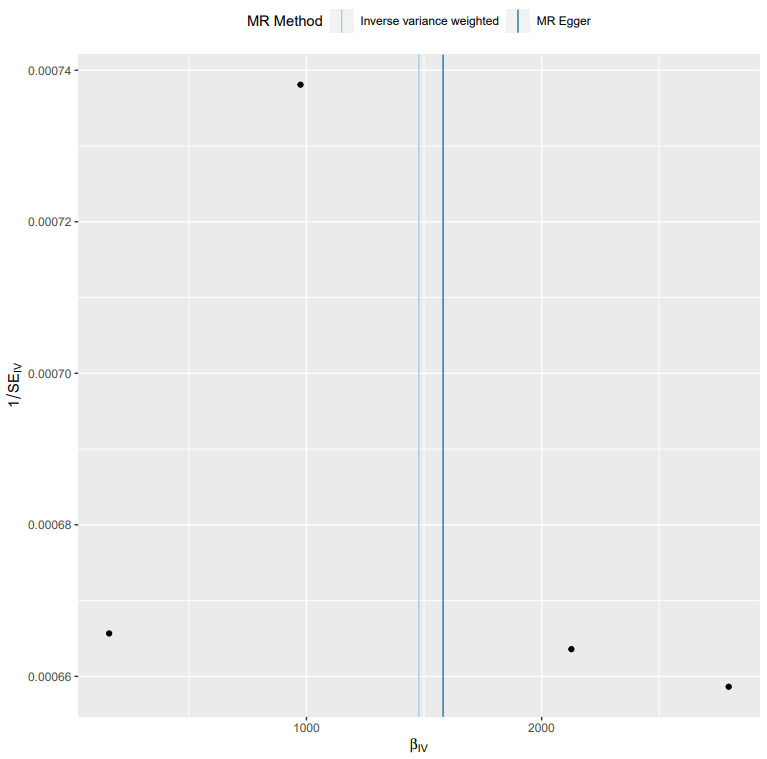

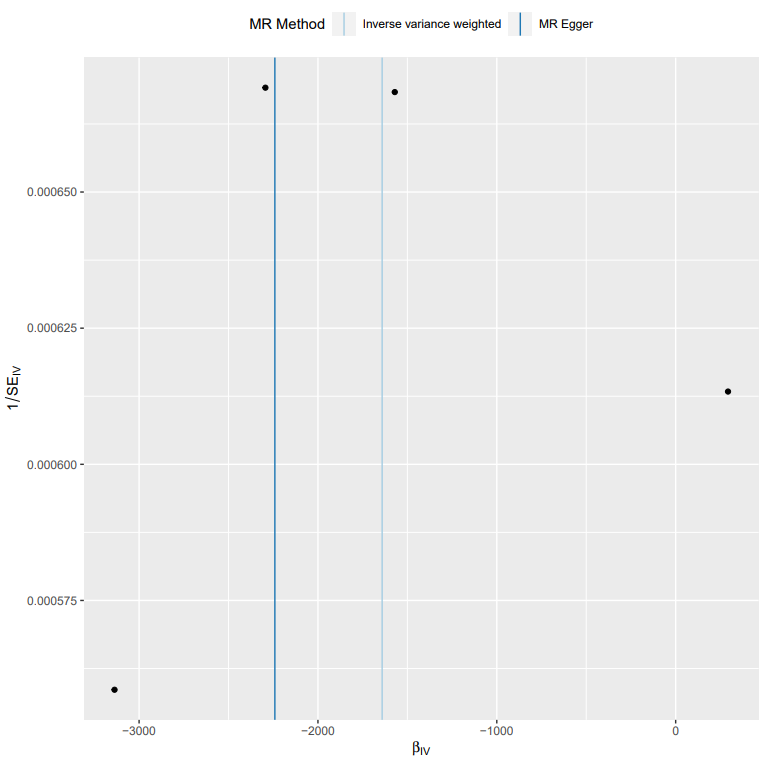


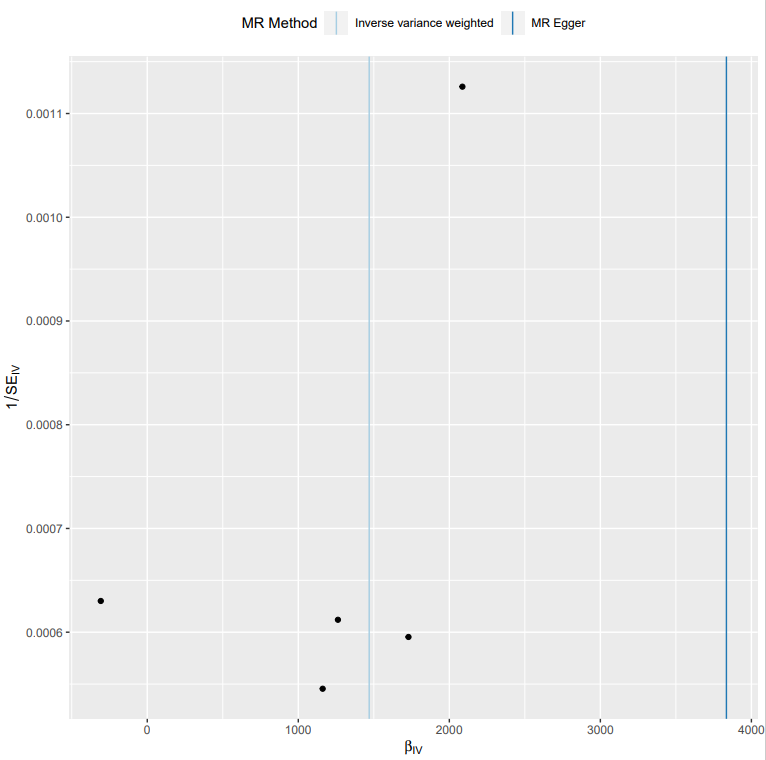

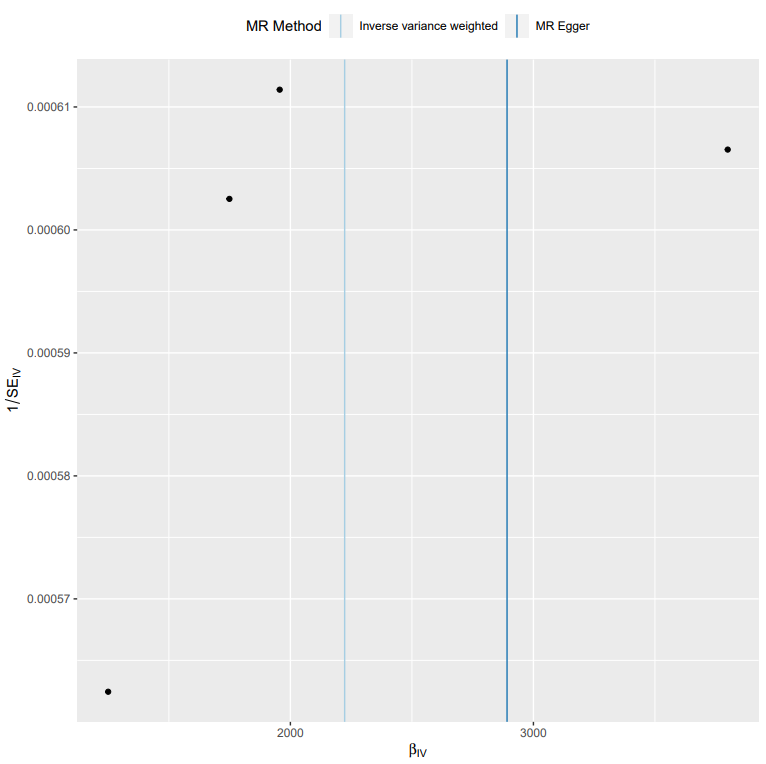


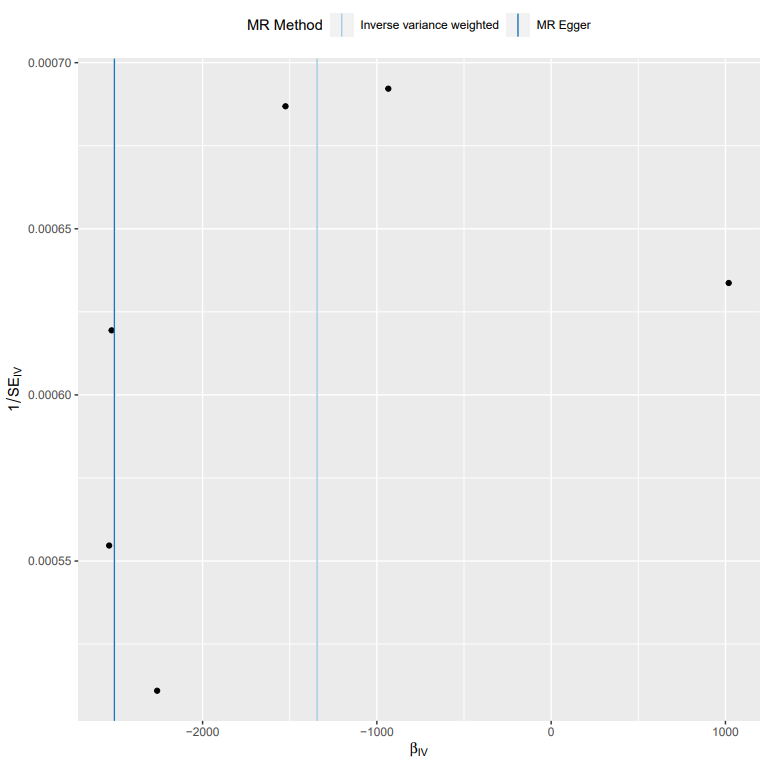

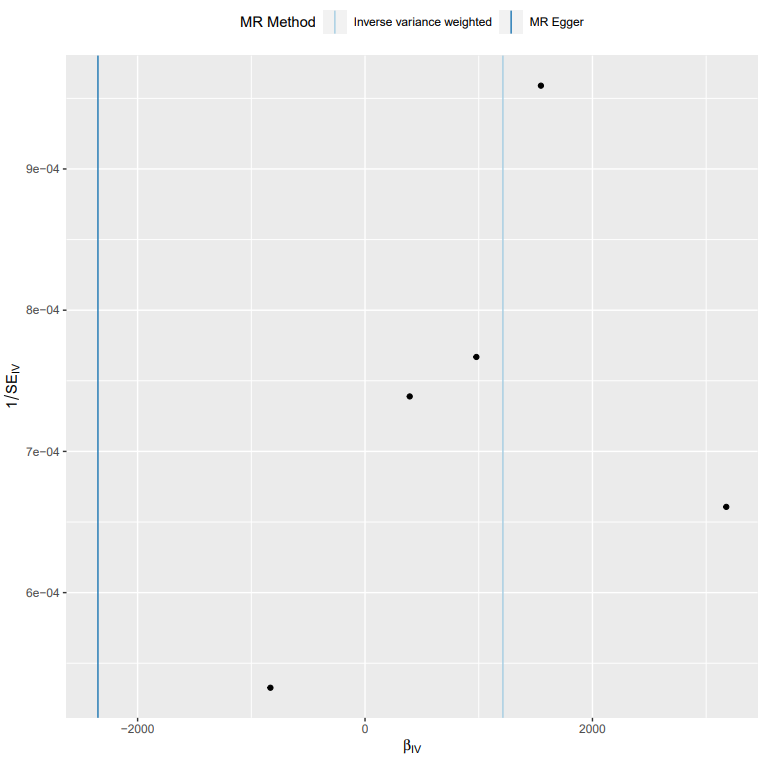


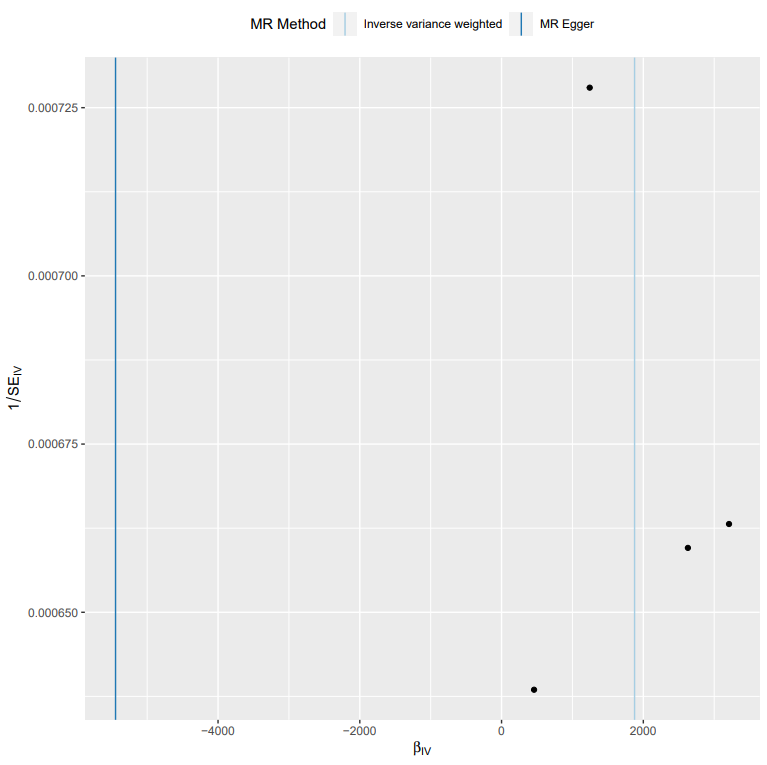

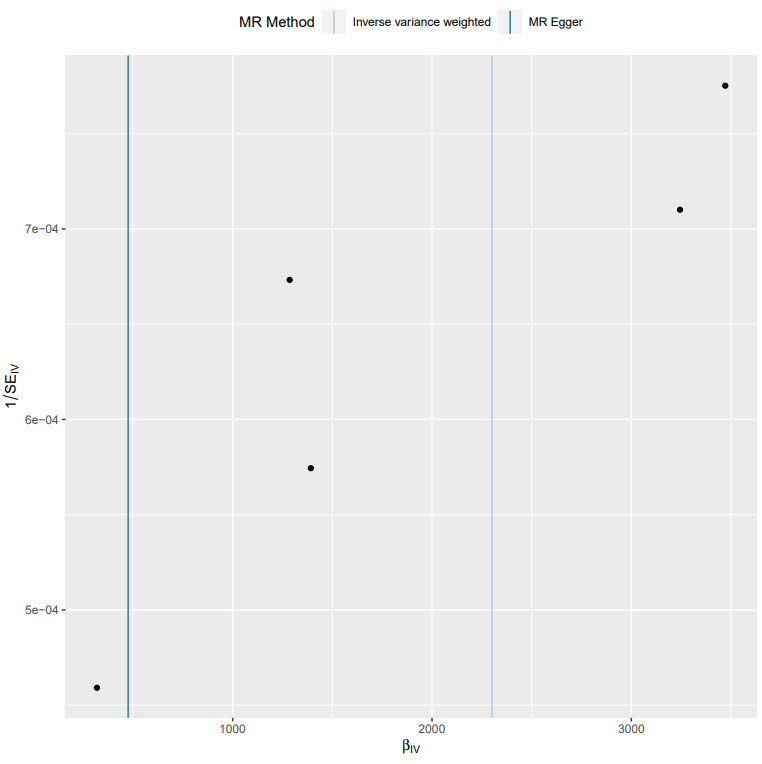


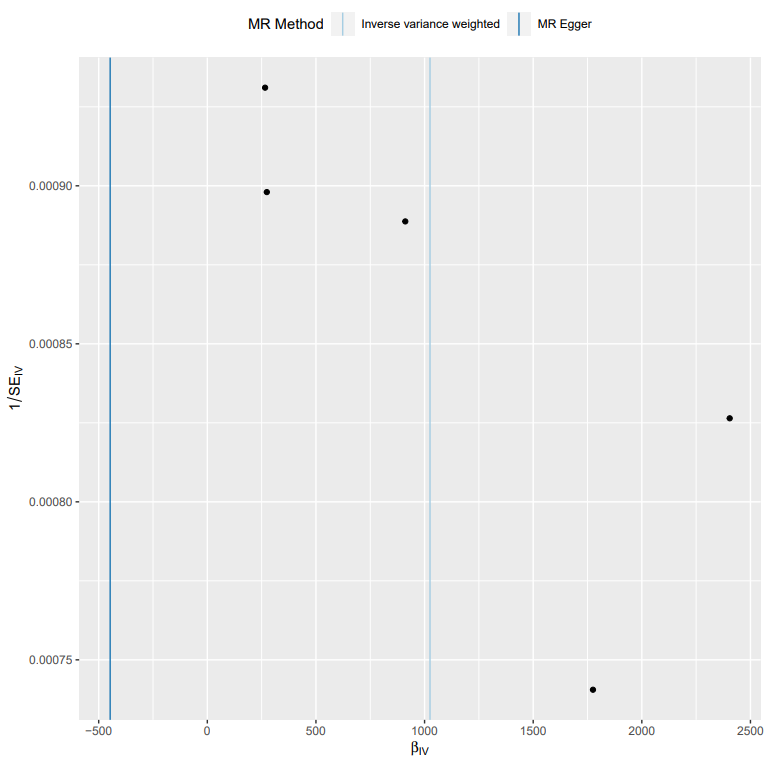


**Fig S3**. Leave-one-out analysis from genetically predicted blood metabolites on brain cortex surficial area.


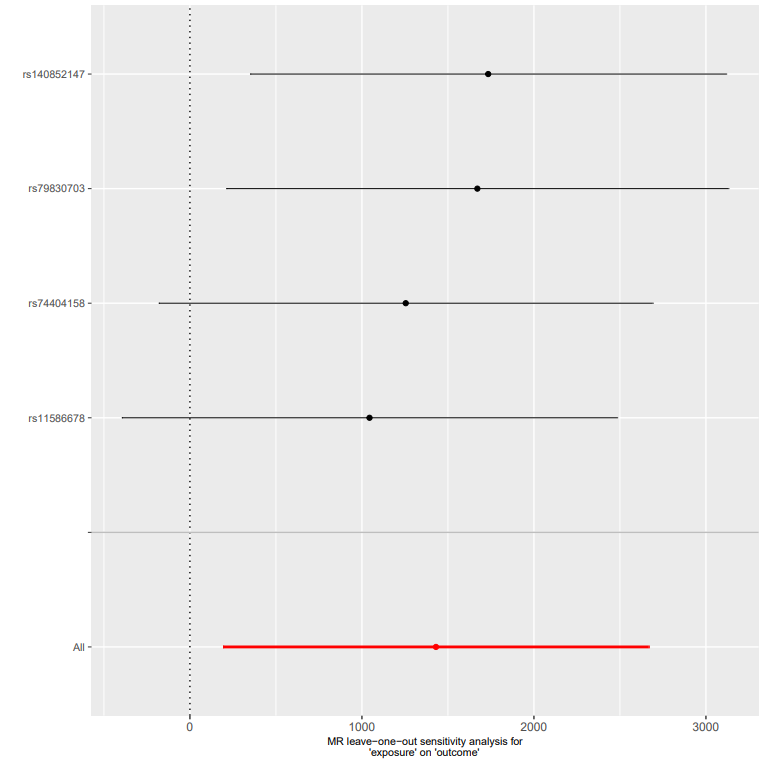

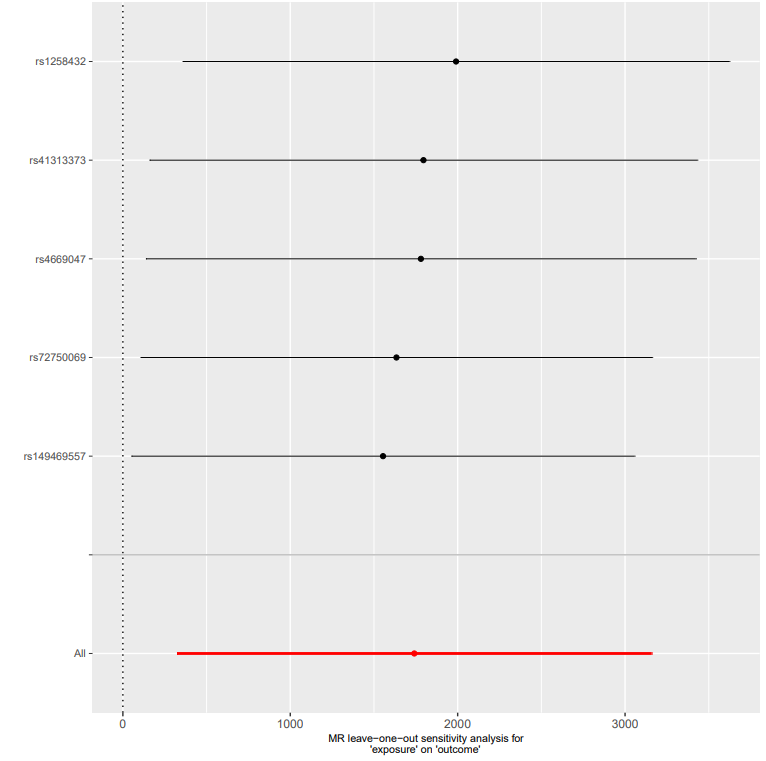


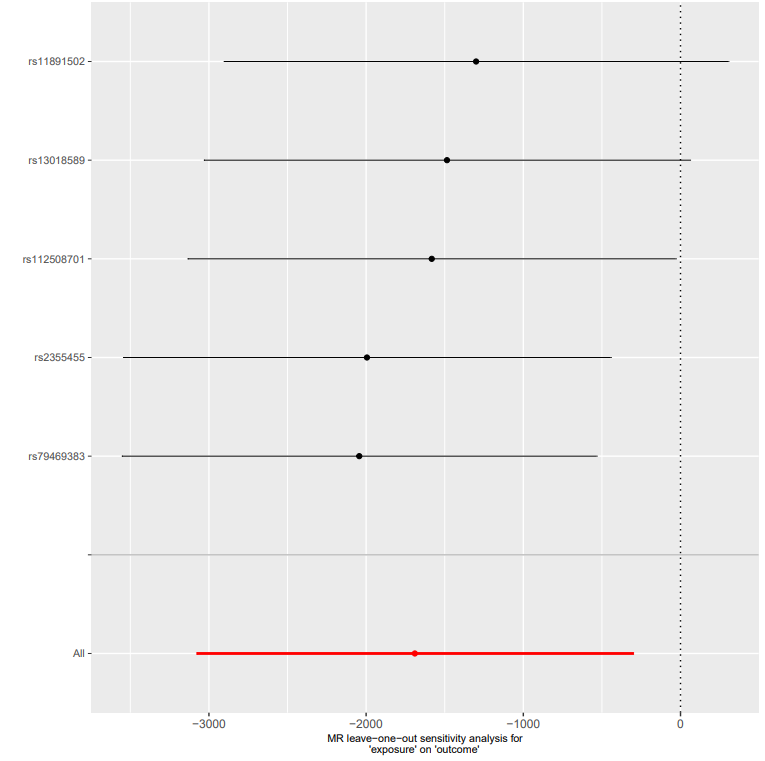

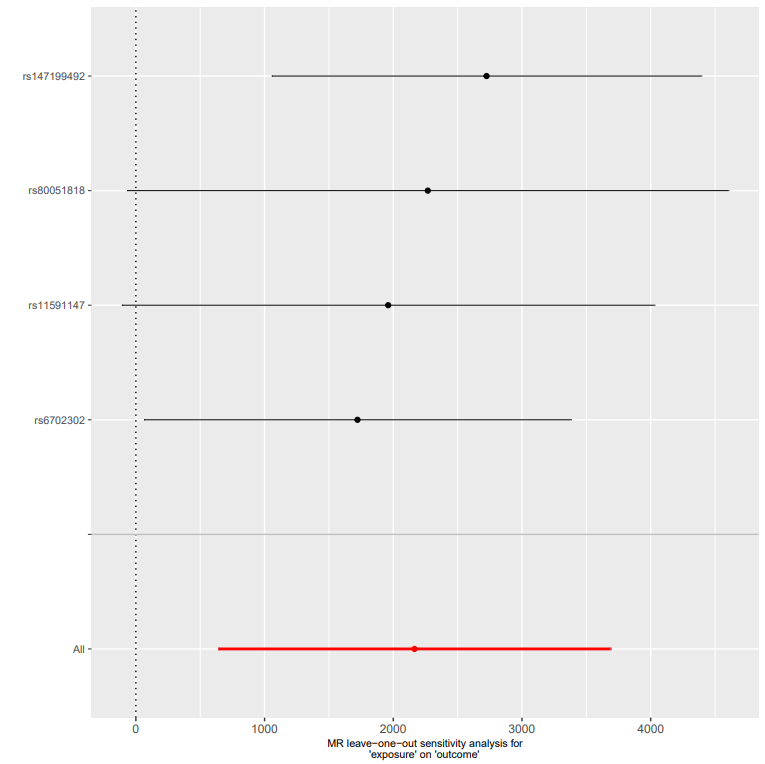


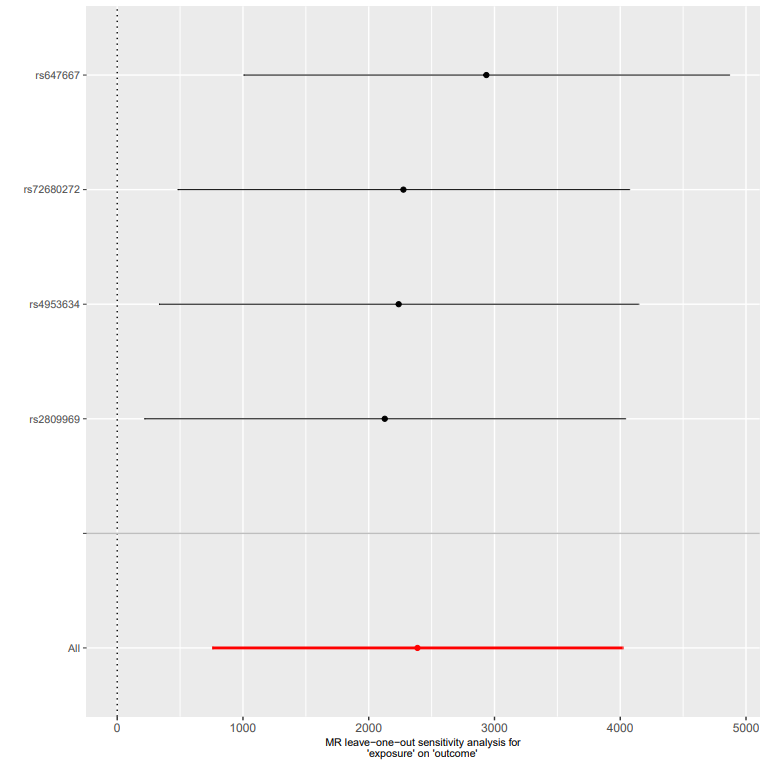

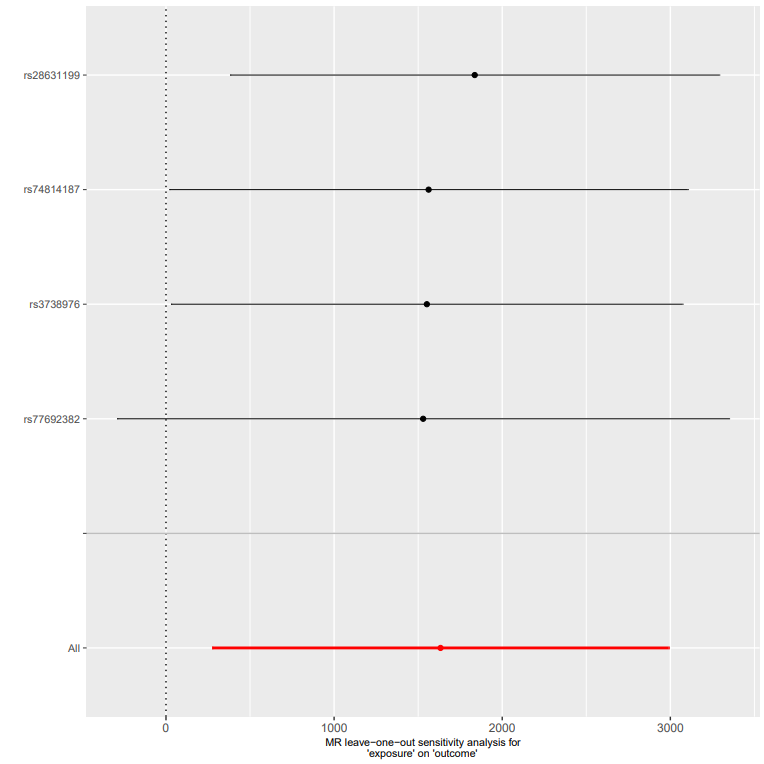


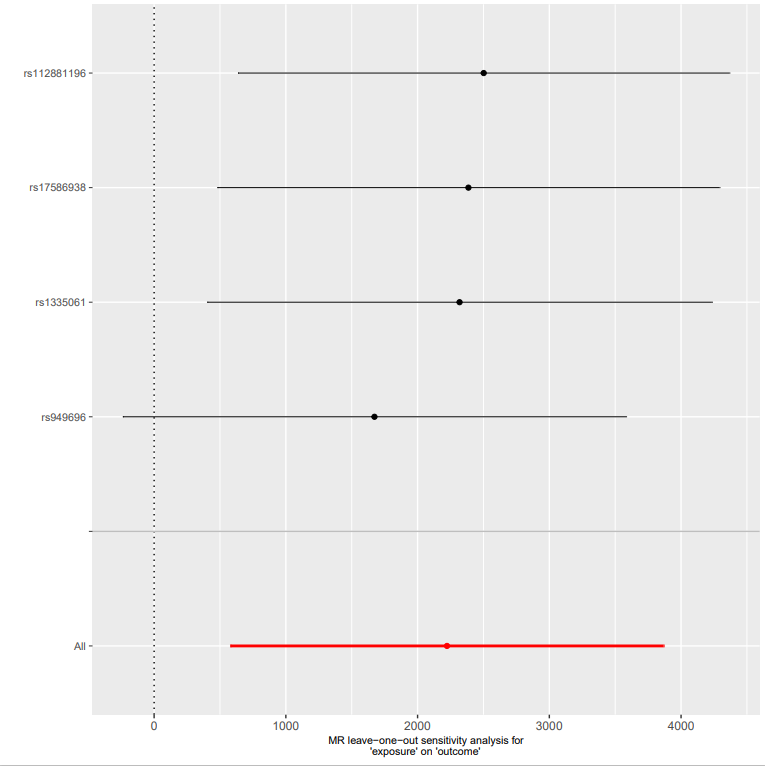

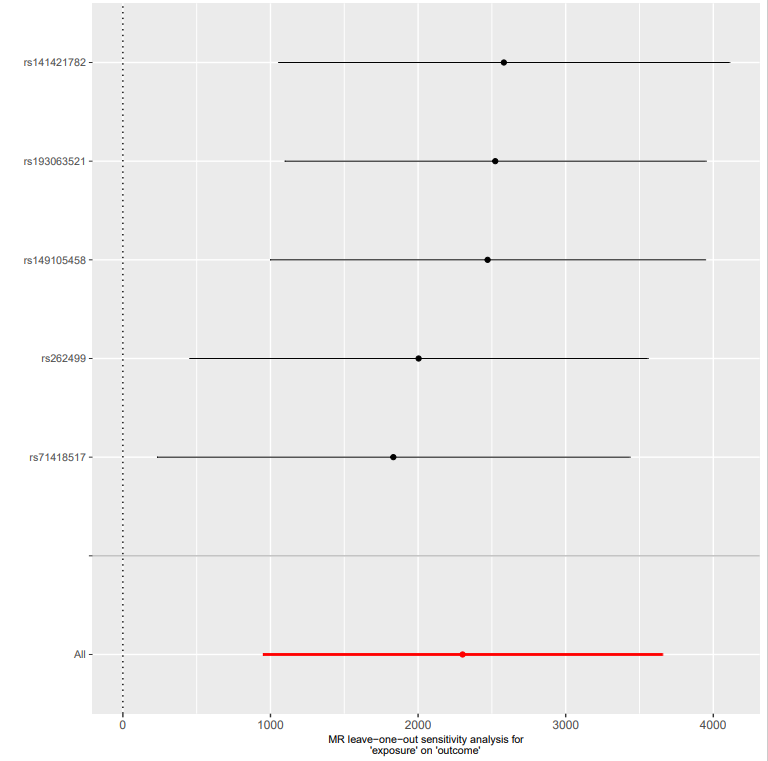


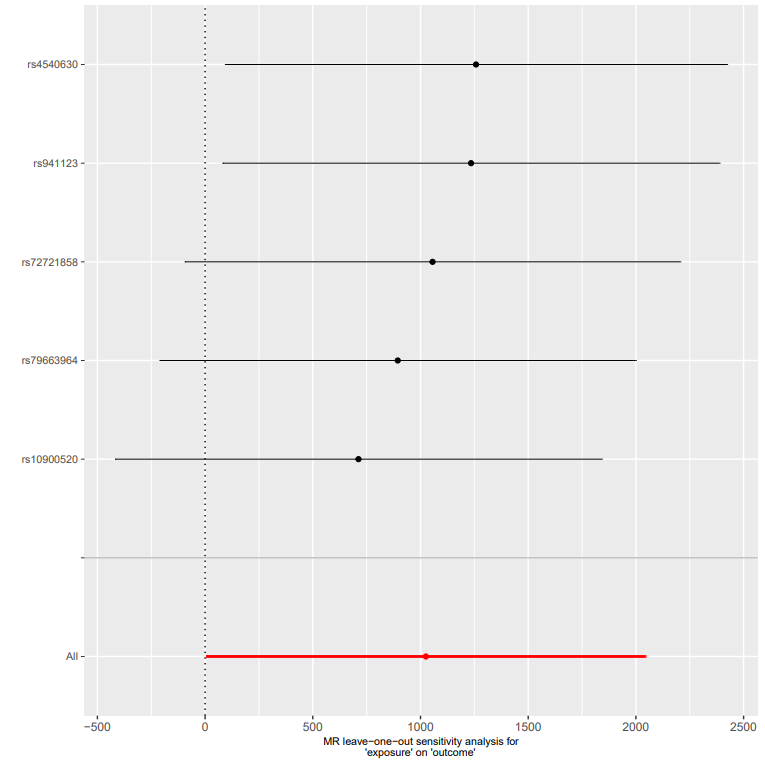


**Fig S4**. Leave-one-out analysis from genetically predicted blood metabolites on brain cortex thickness.


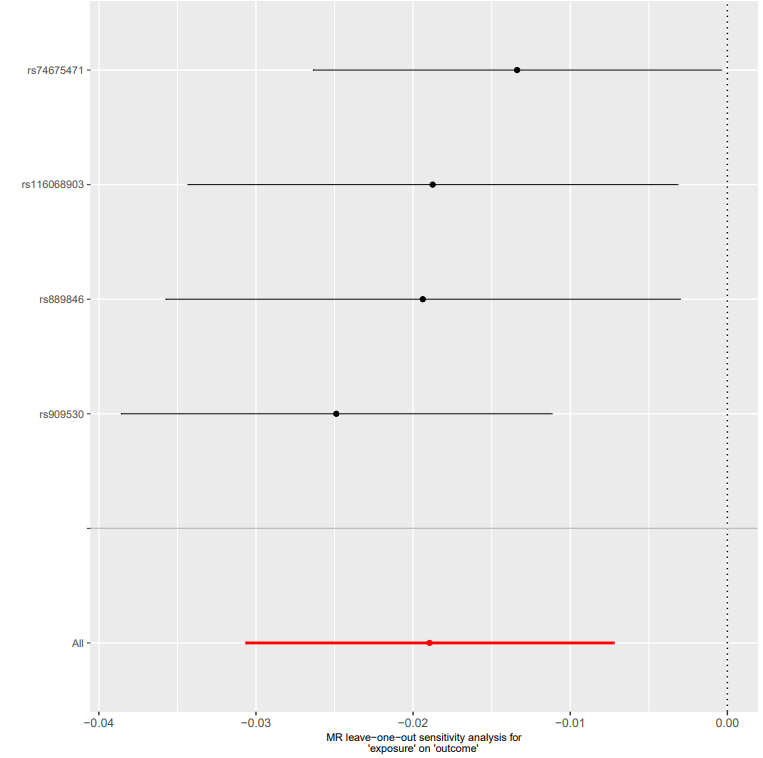

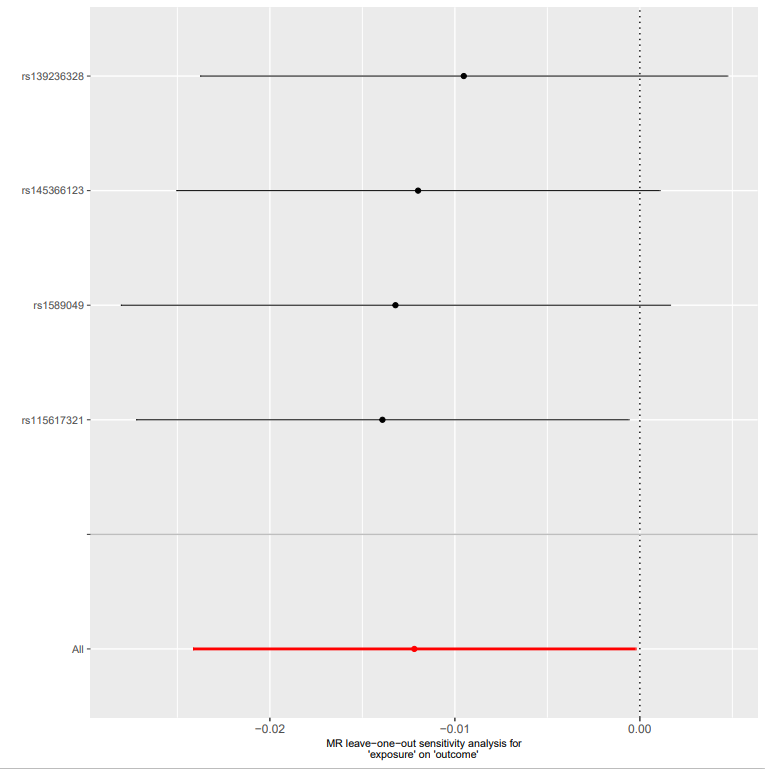


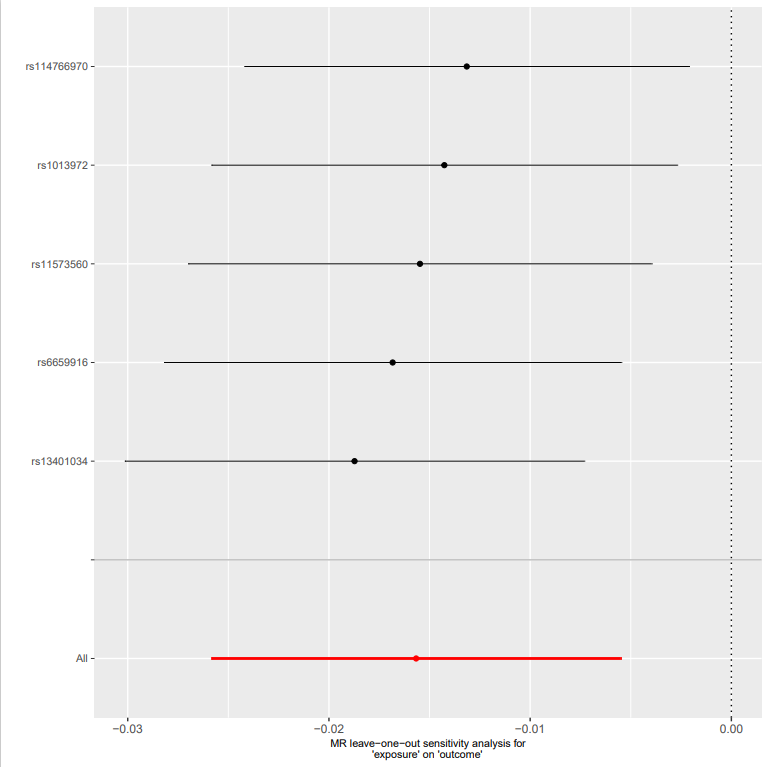

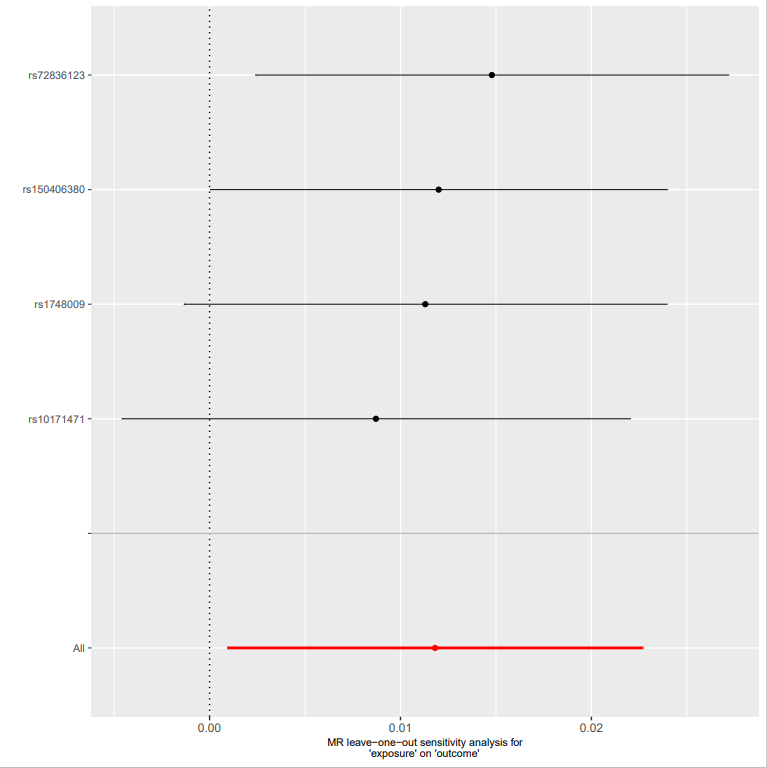


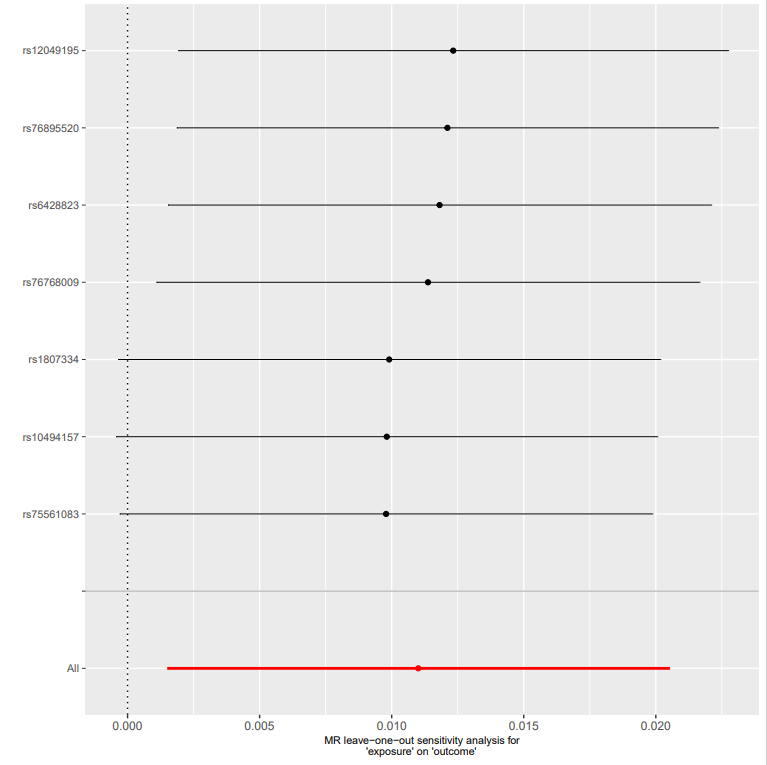

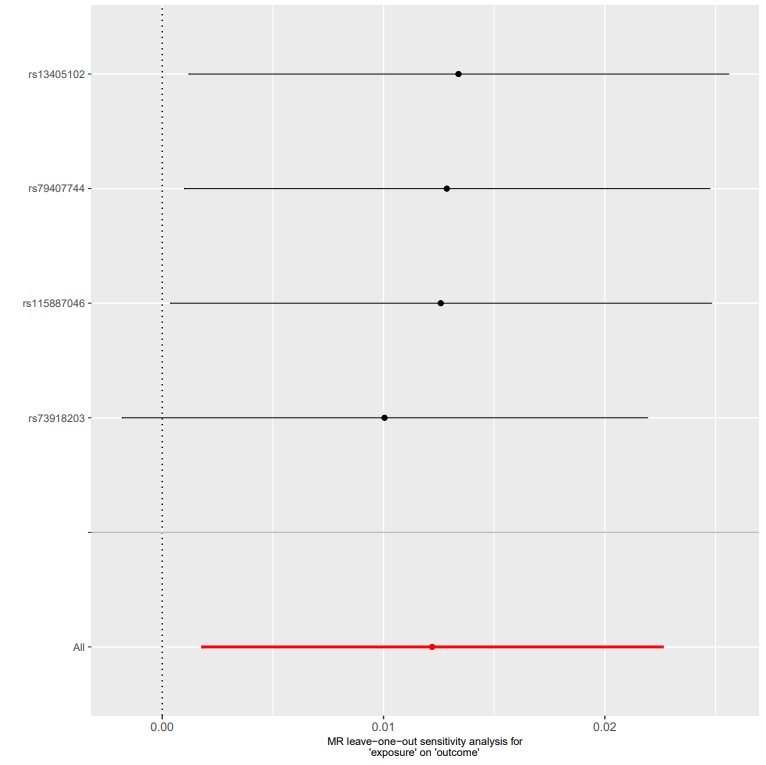


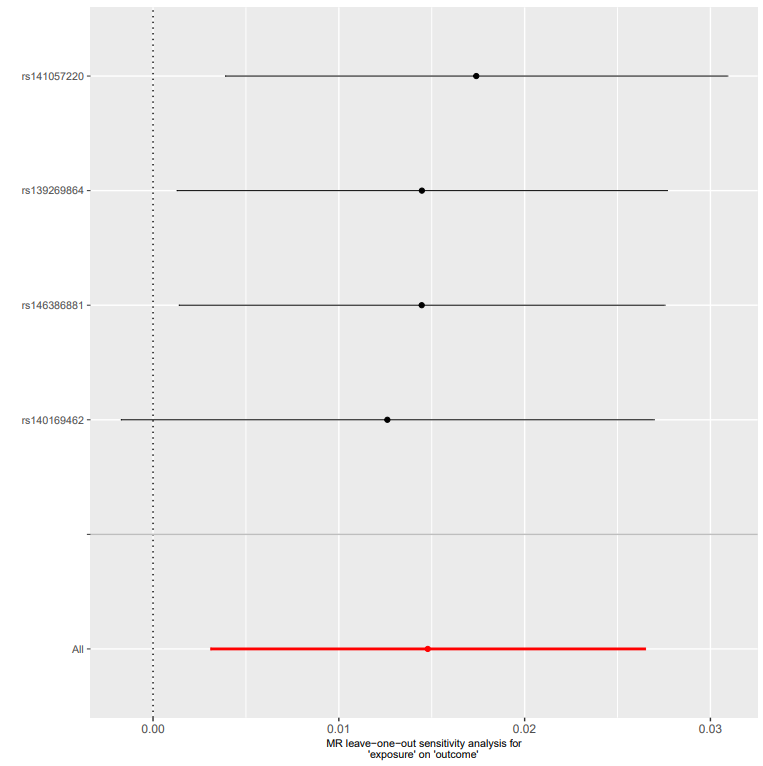

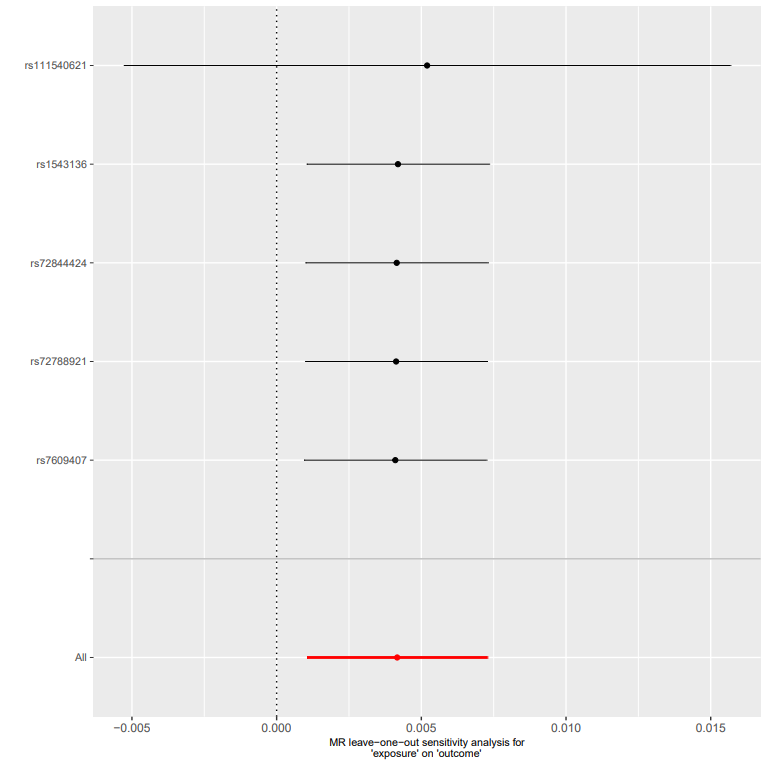


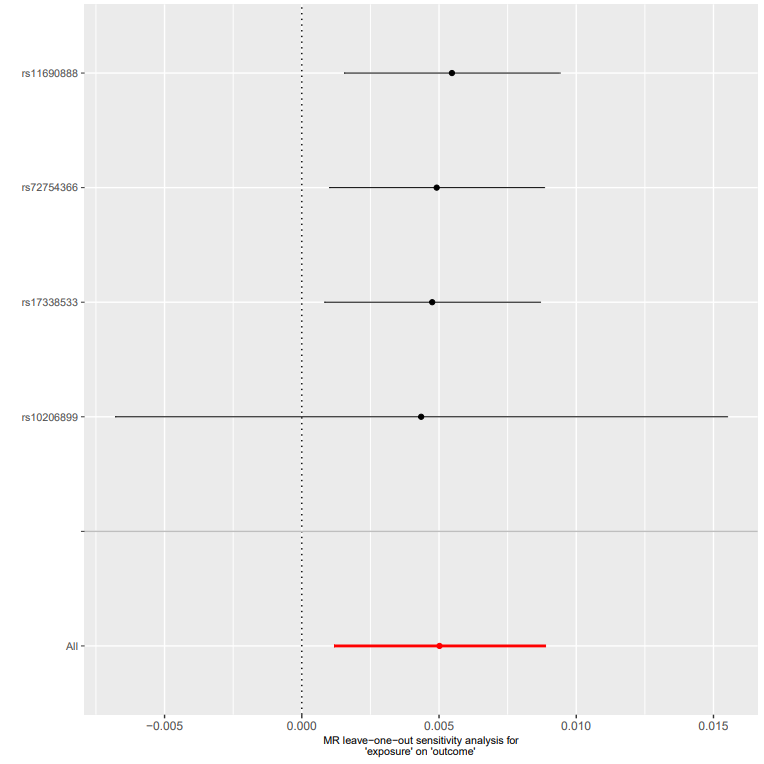

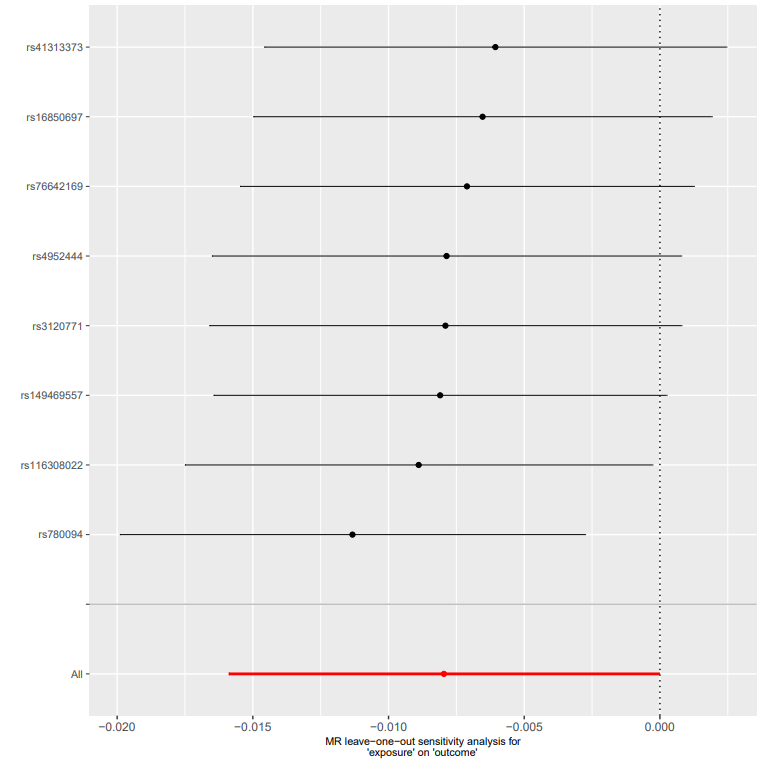


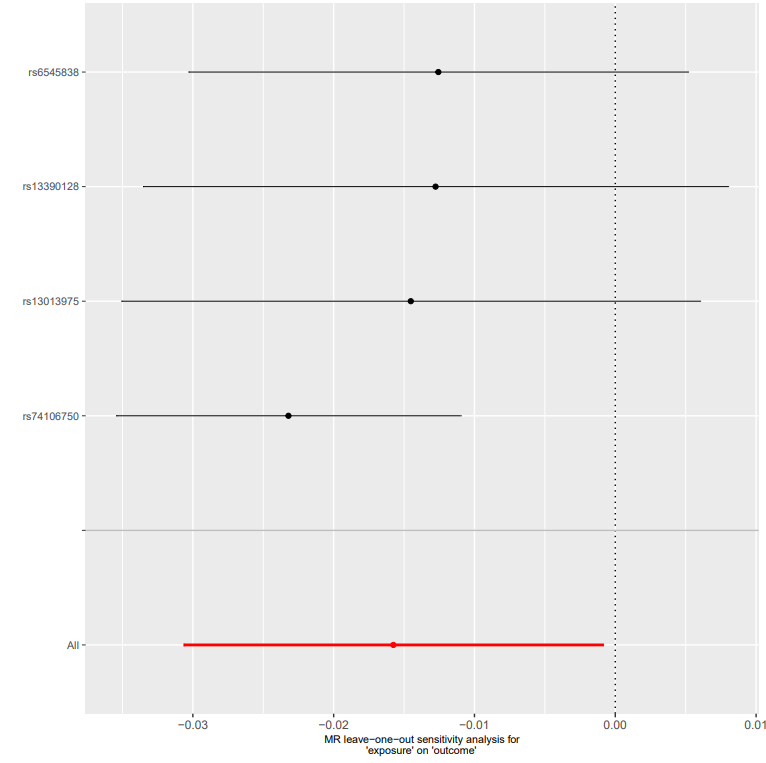

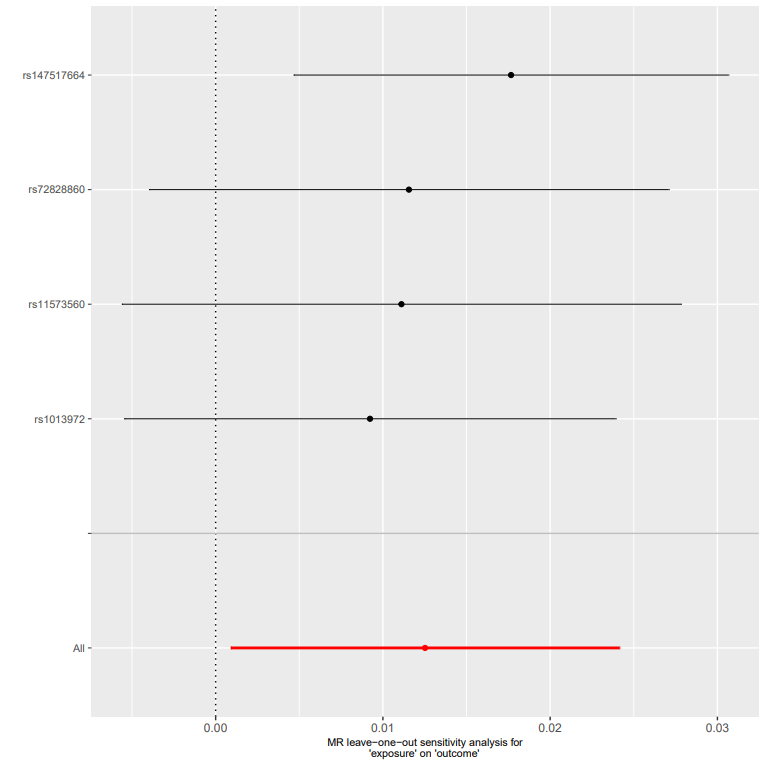


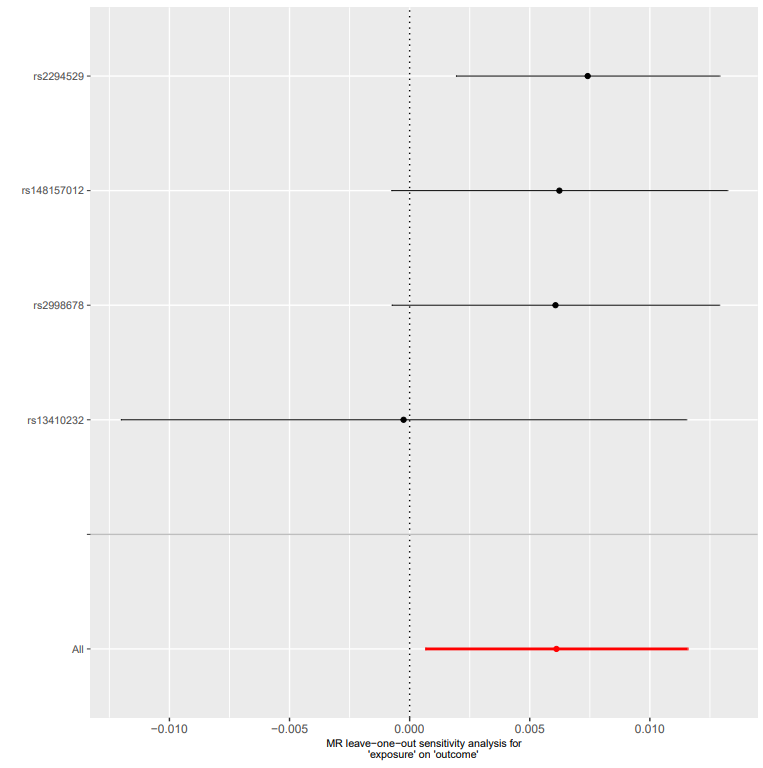


**Fig S5**. Scatter plots from genetically predicted blood metabolites on brain cortex thickness.


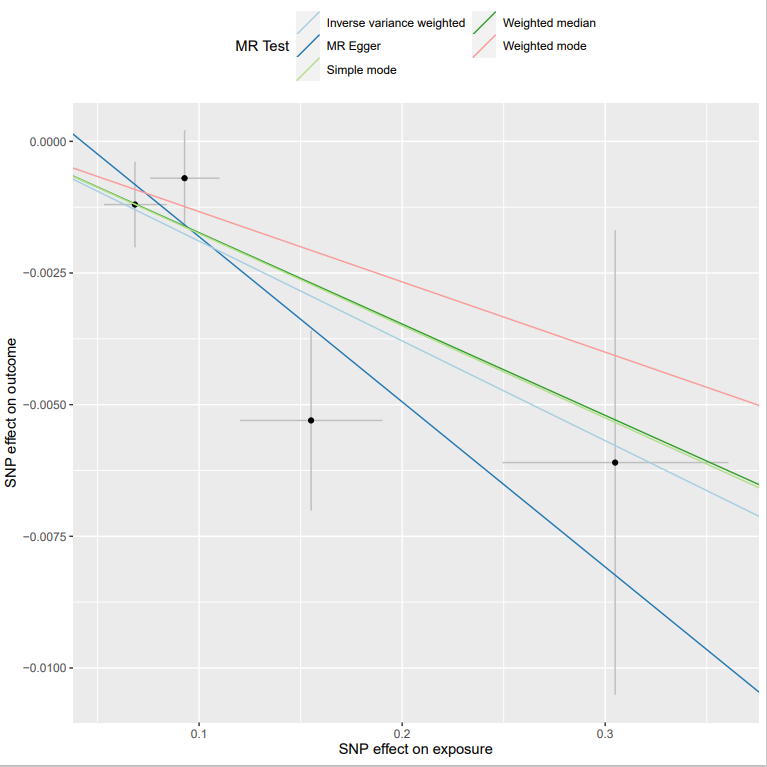

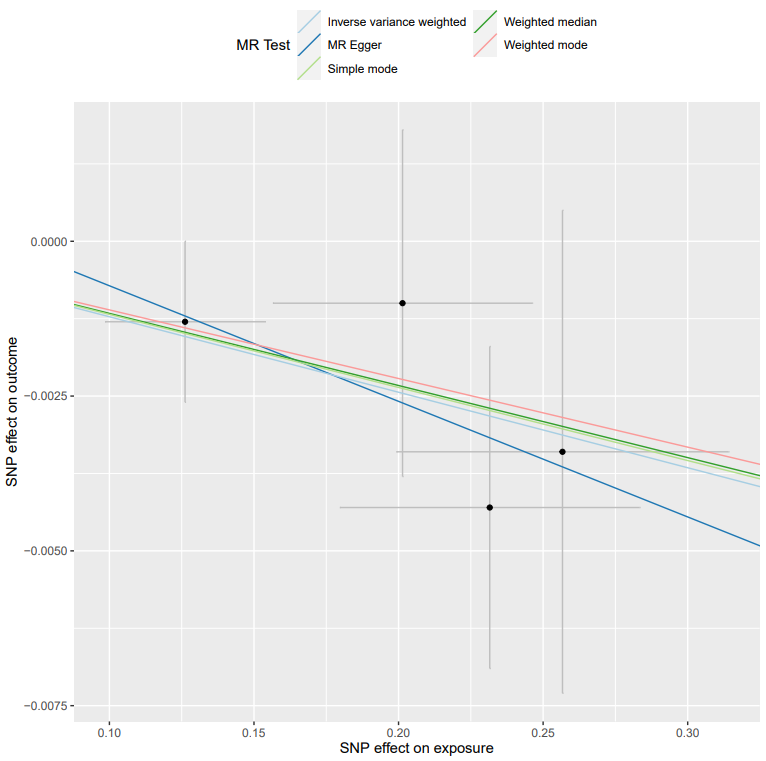


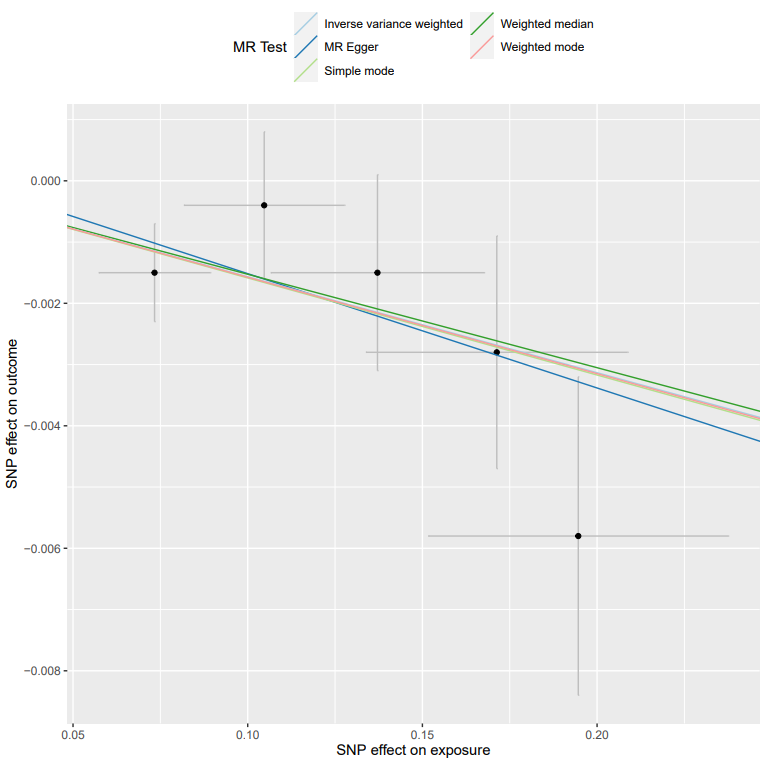

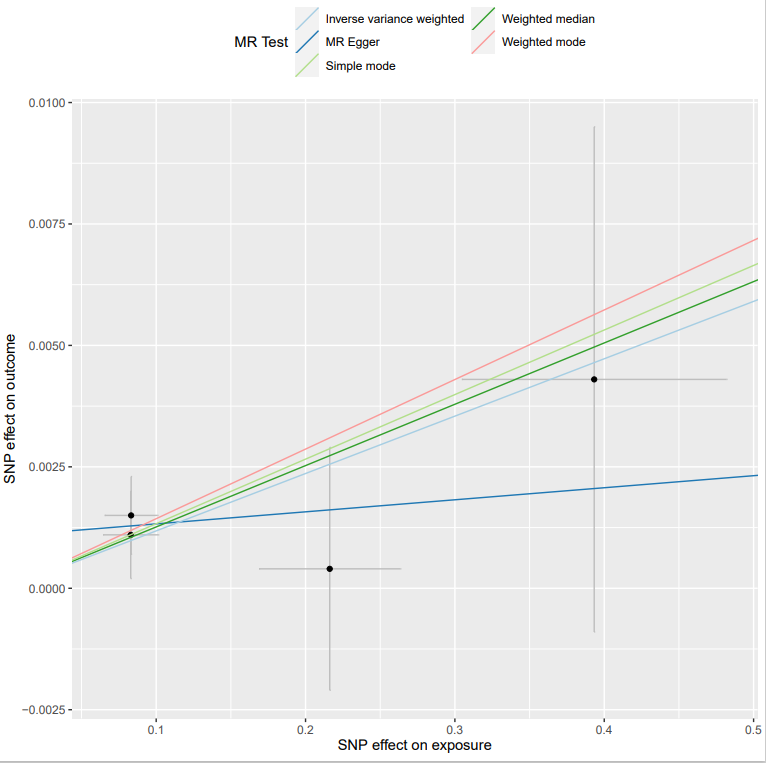


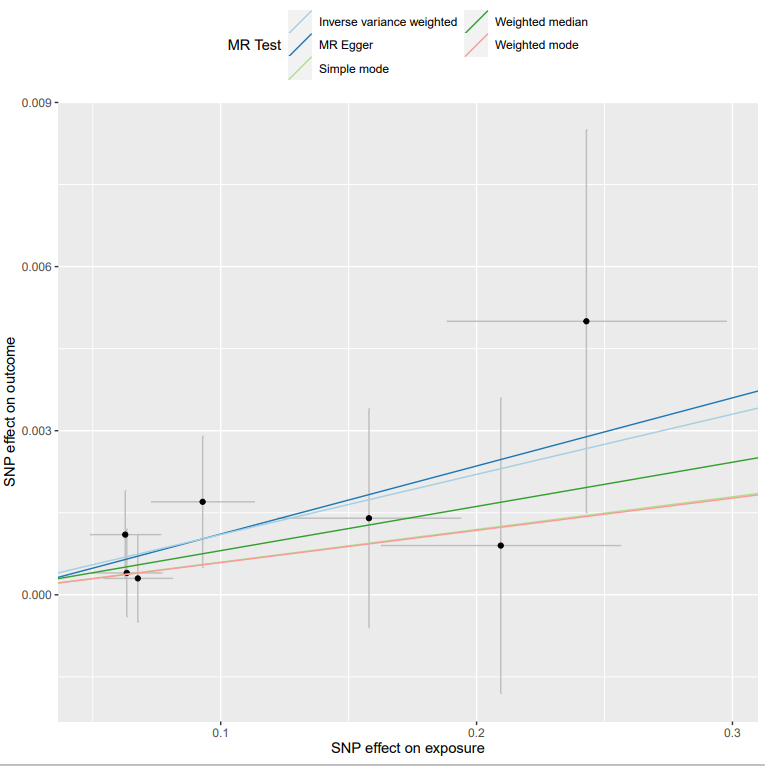

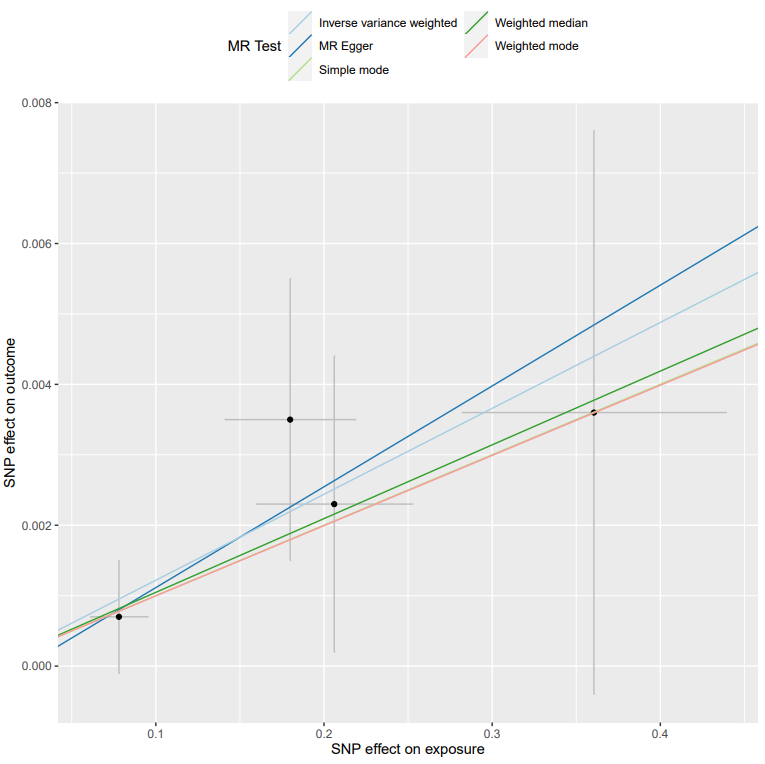


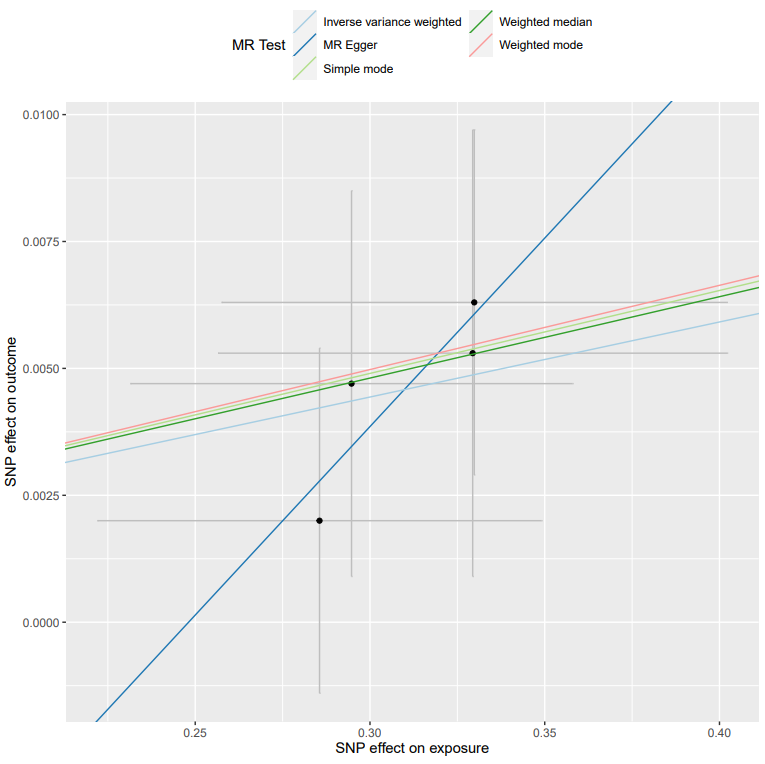

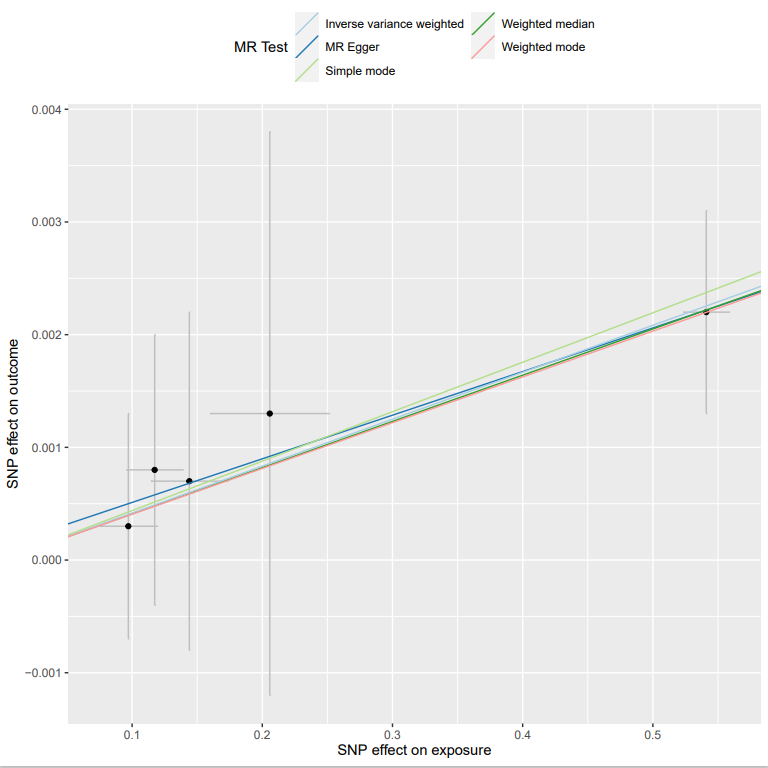


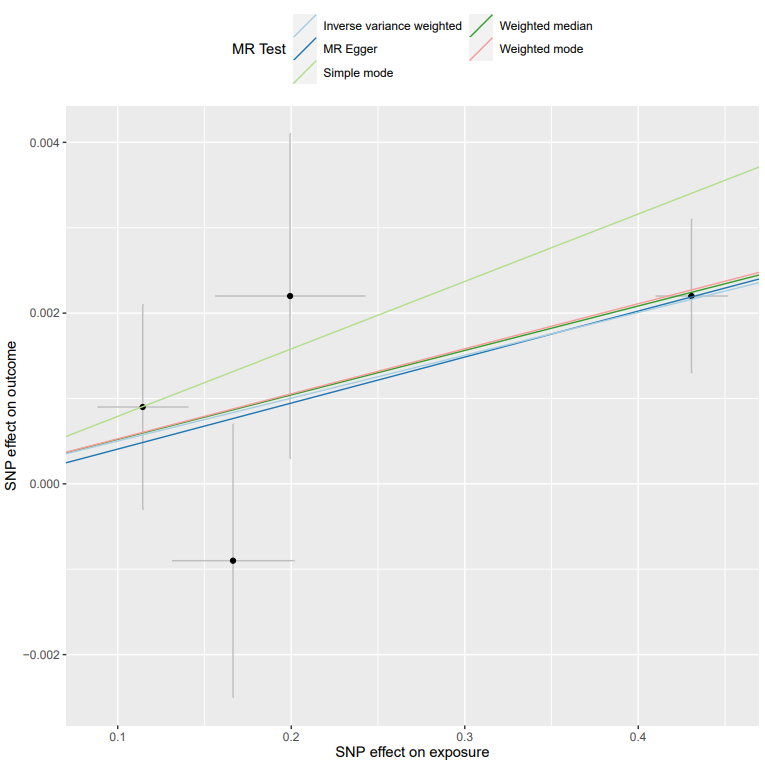

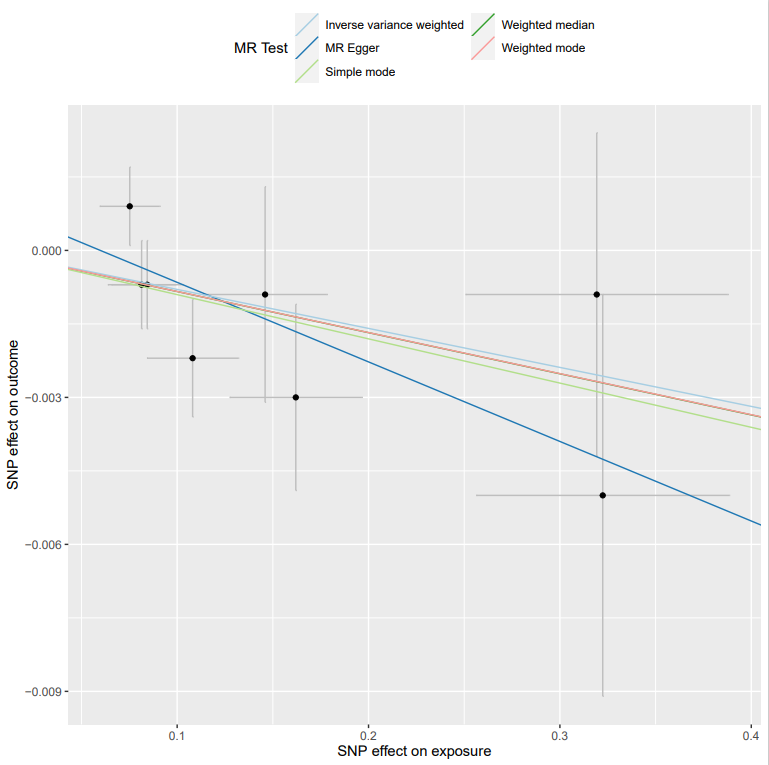


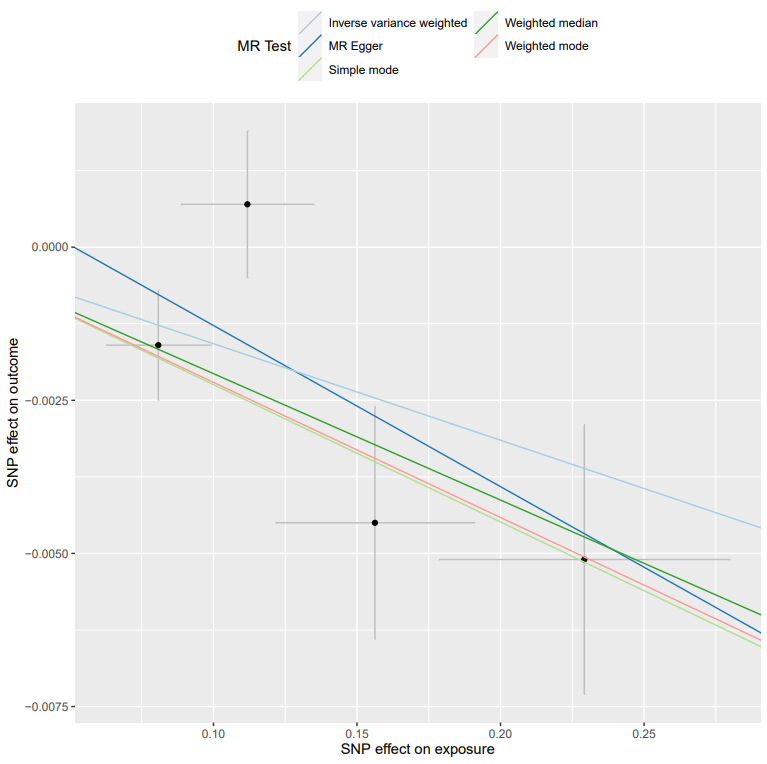

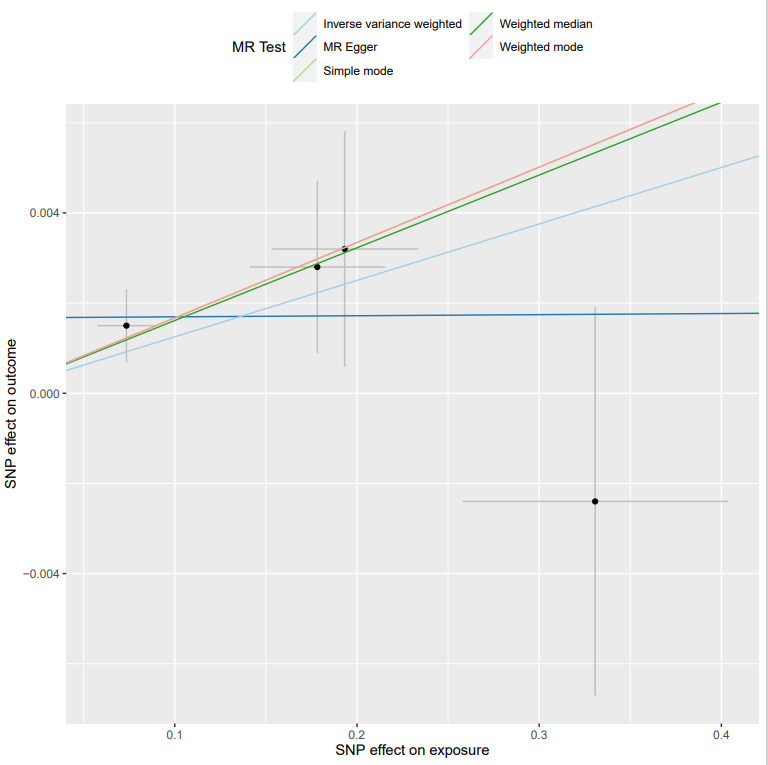


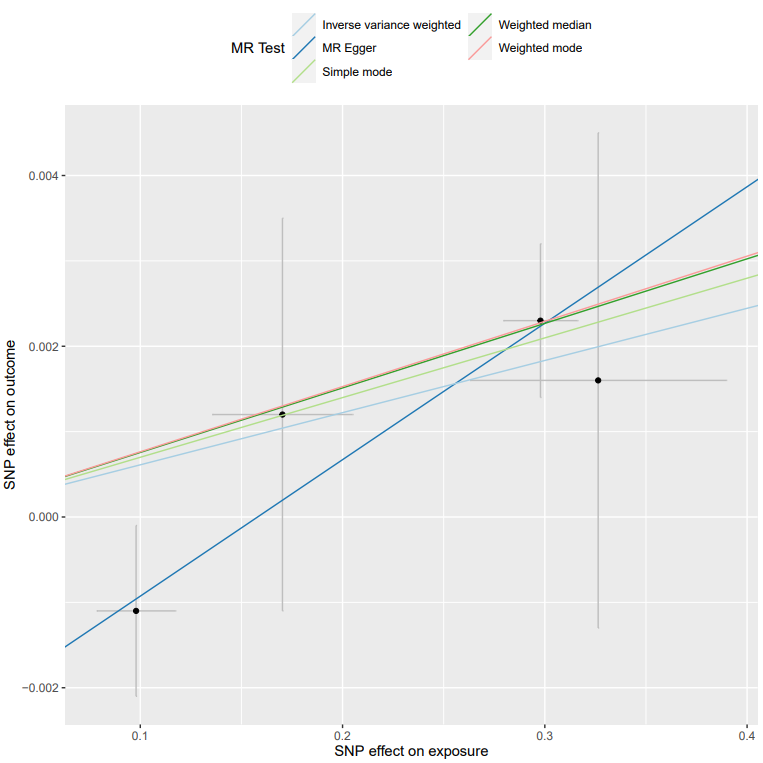


**Fig S6**. funnel plots from genetically predicted blood metabolites on brain cortex thickness.


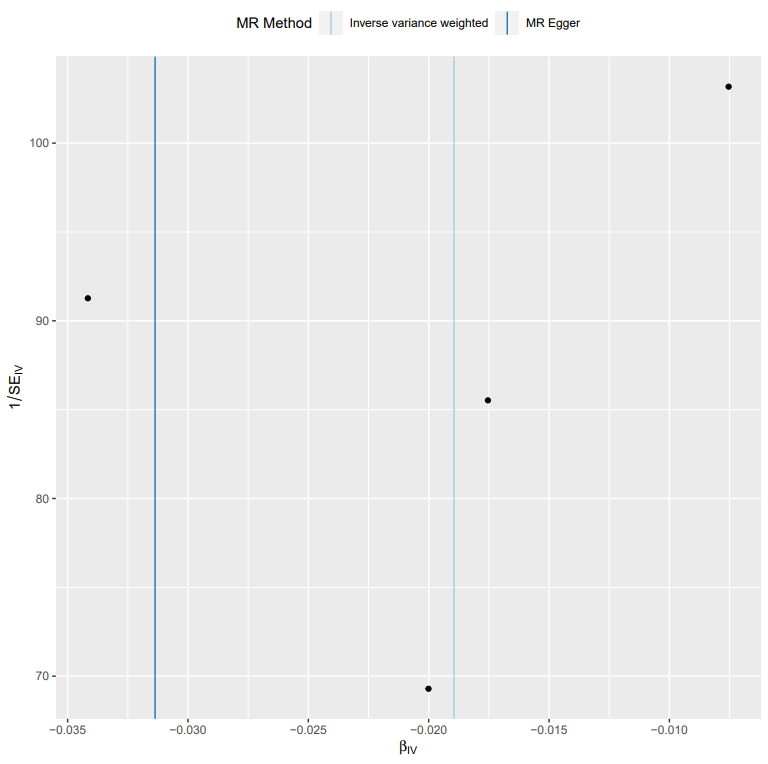

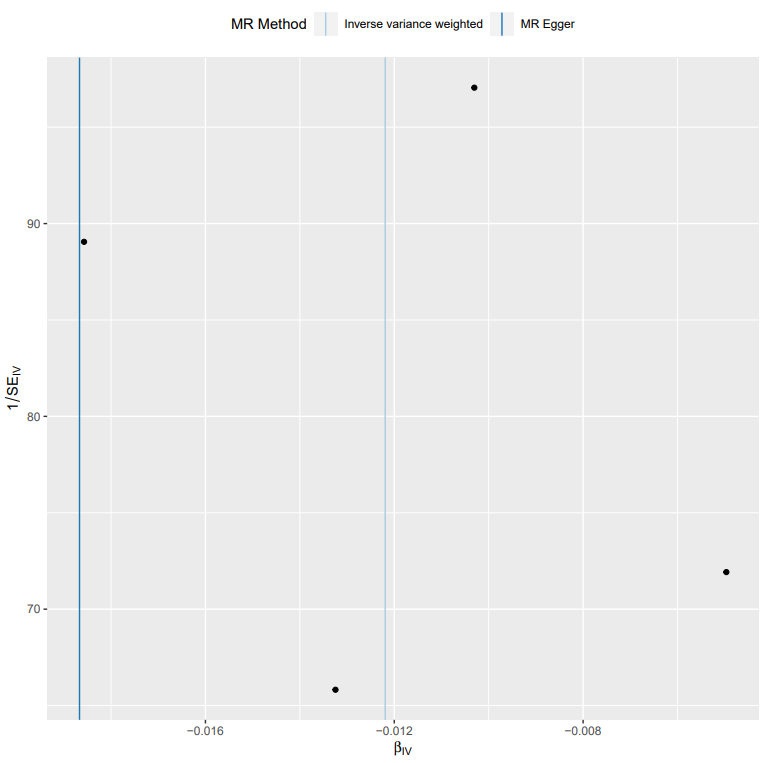


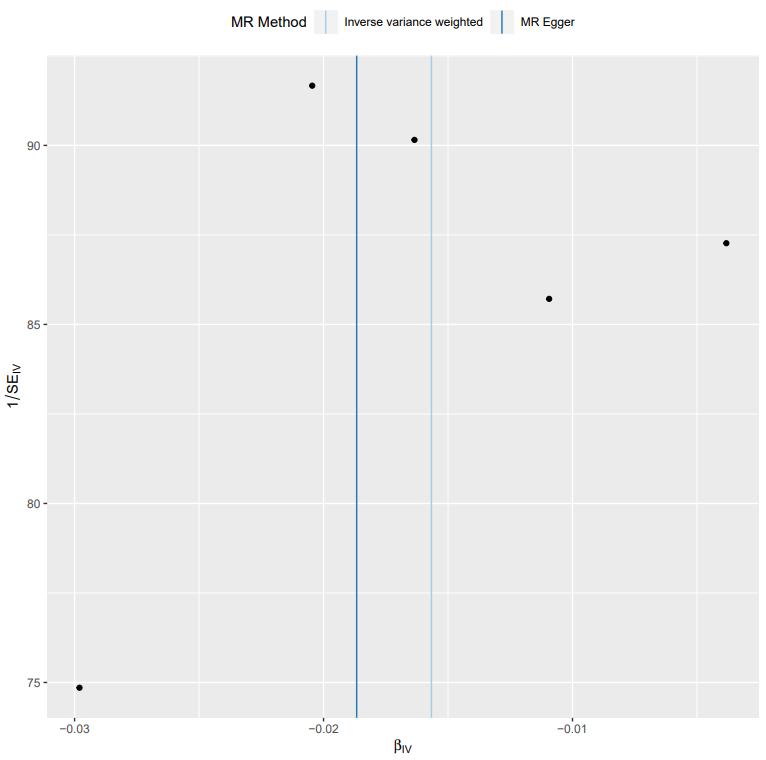

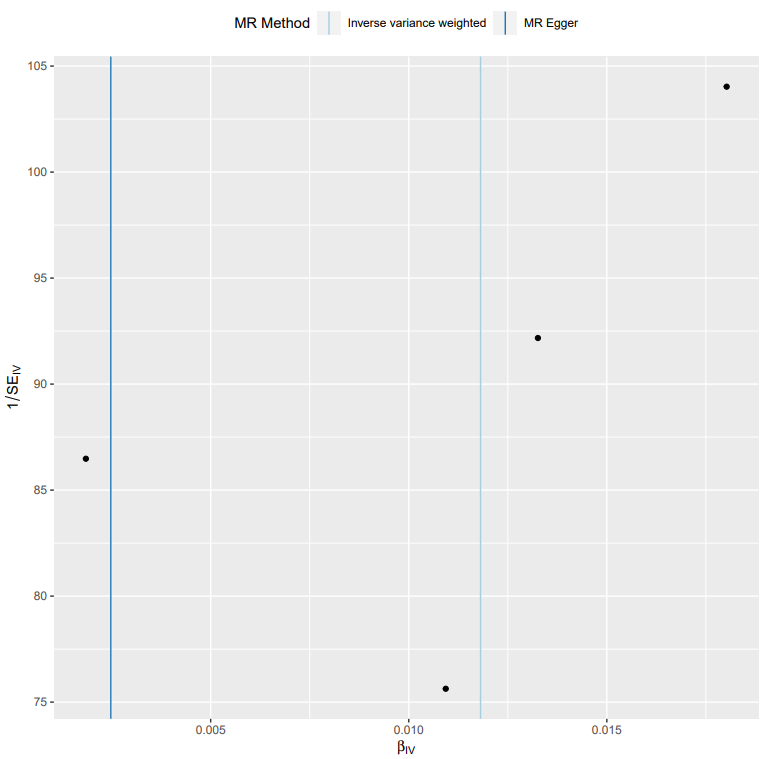


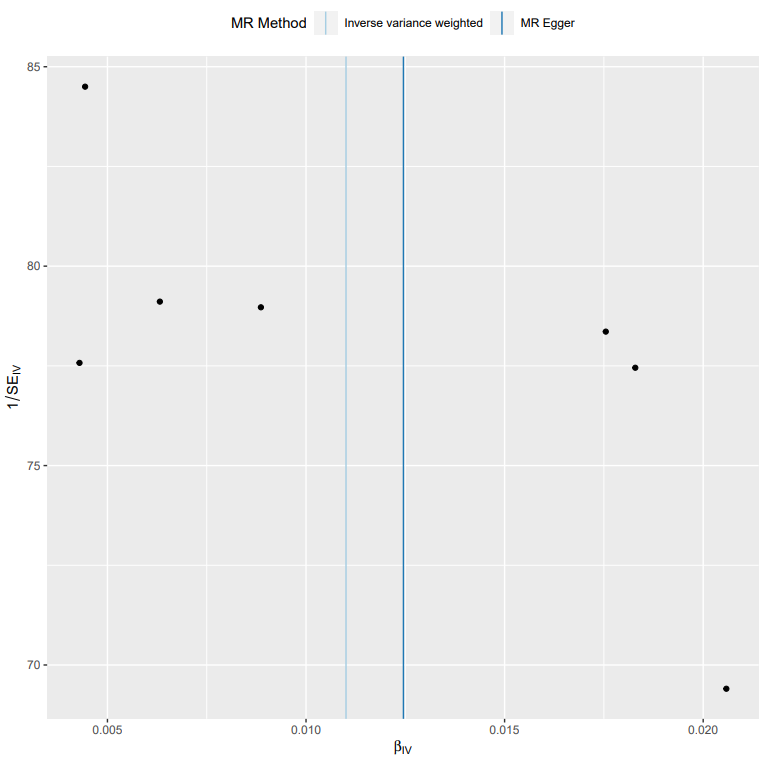

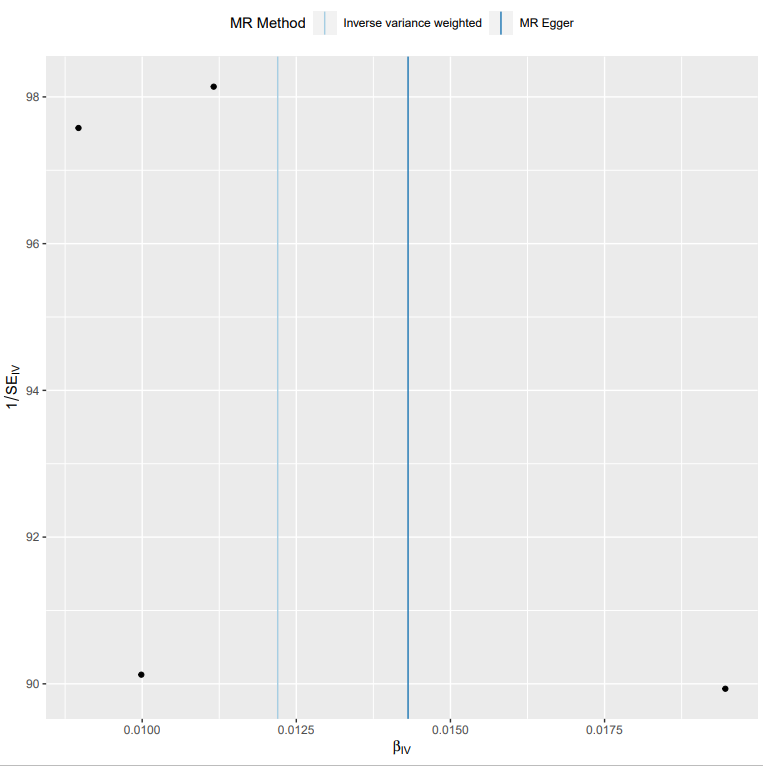


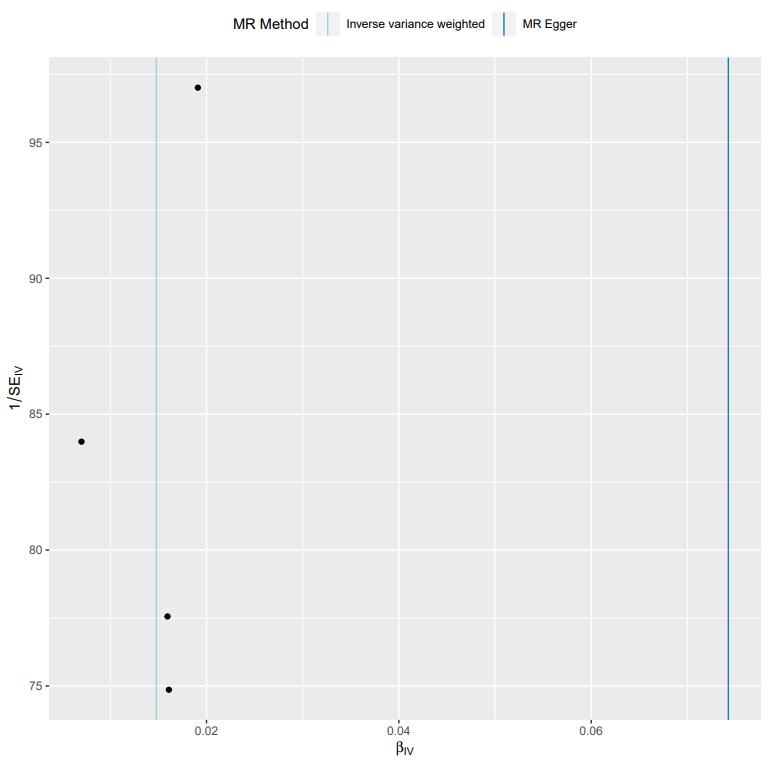

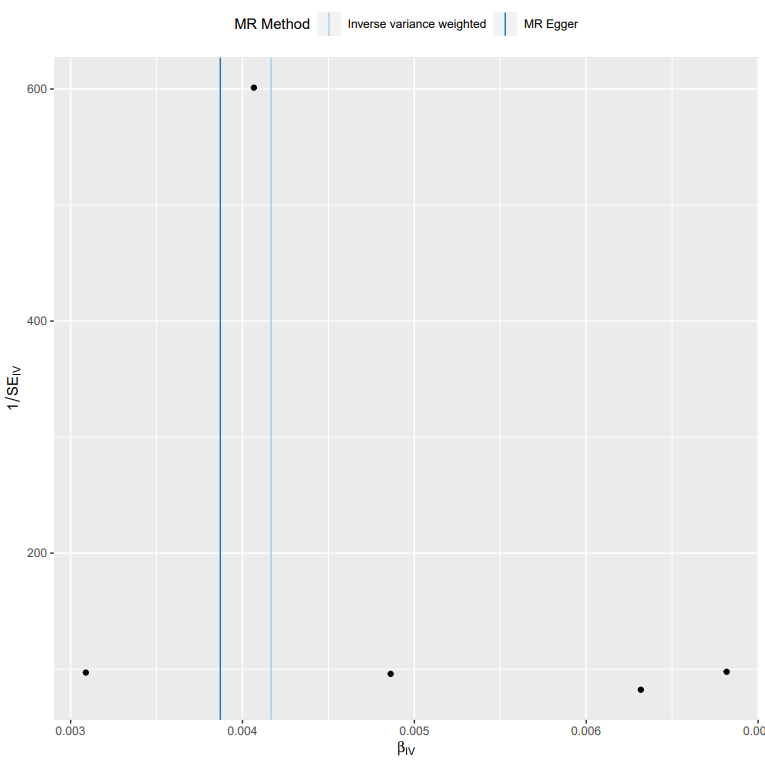


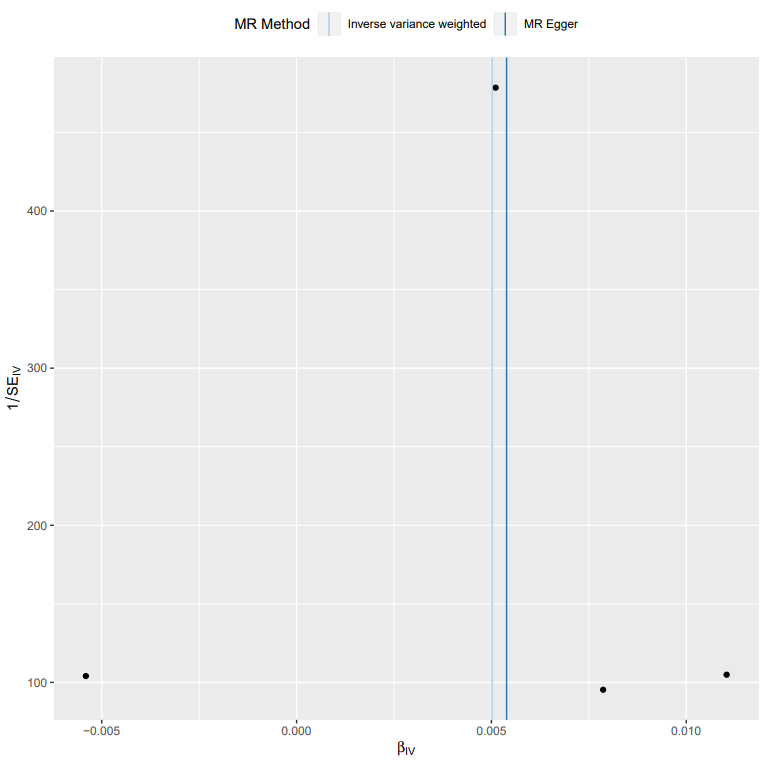

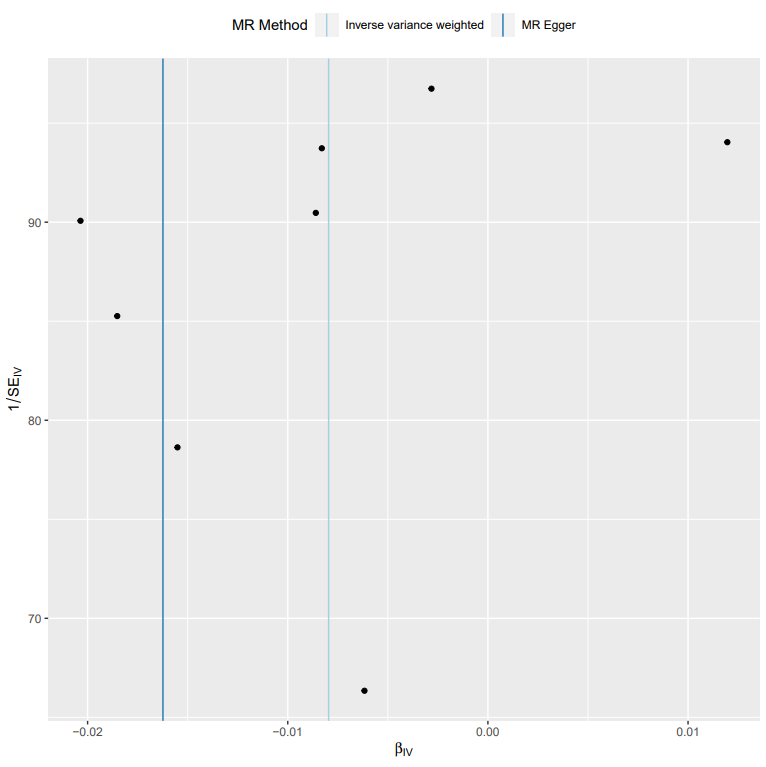


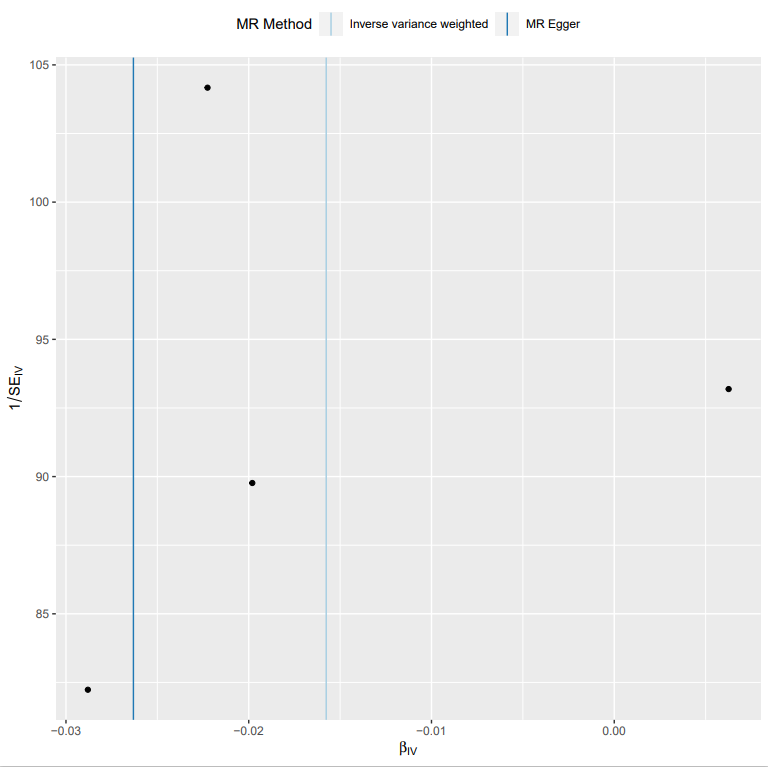

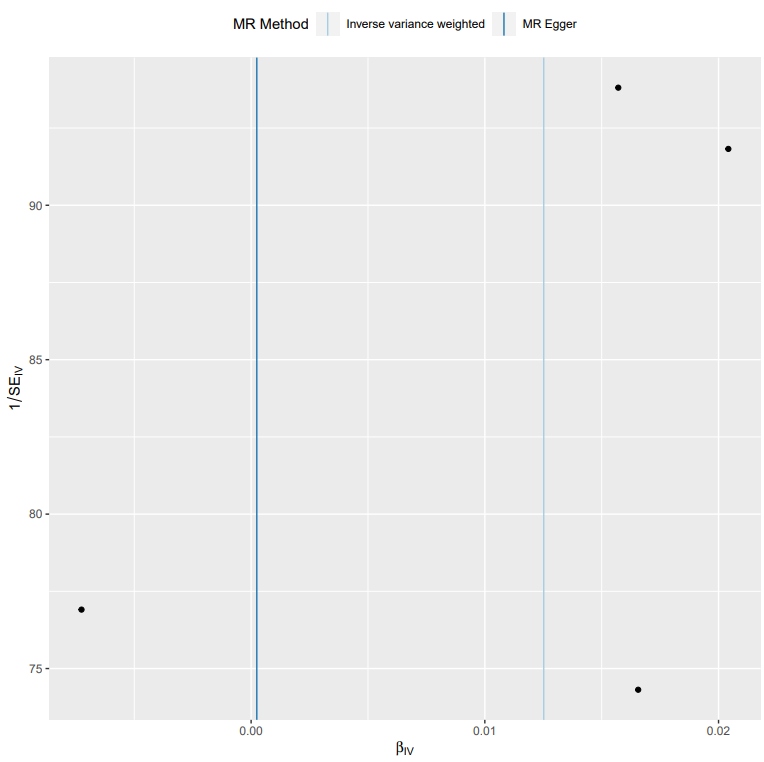


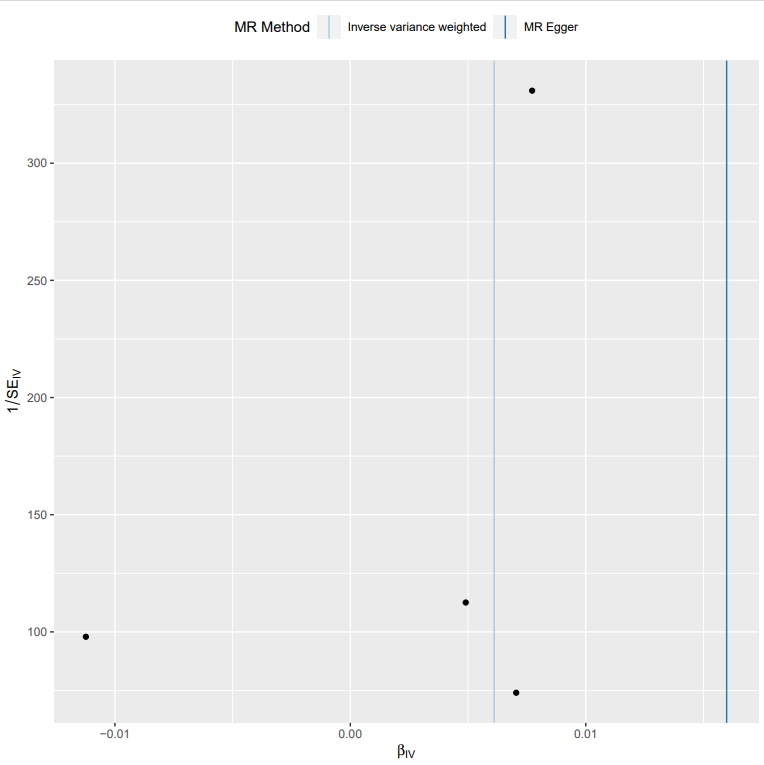

Supplement: Supplementary file 2 [file Table_2.docx]
